# S-Ketamine Preserves Oxidative Metabolism and Reduces Metabolic Stress During Neuronal Hyperexcitability

**DOI:** 10.3390/ijms27188220

**Published:** 2026-09-15

**Authors:** Georg Riepe, Jonas Schunack, Jörg Rösner, Iwona Wallach, Nikolaus Berndt, Agustin Liotta

**Affiliations:** 1 Department of Molecular Toxicology, German Institute of Human Nutrition Potsdam-Rehbruecke (DIfE), Arthur-Scheunert-Allee 114-116, 14558 Nuthetal, Germany; 2 Department of Radiology, Charité—University Medical Center Berlin, Corporate Member of Freie Universität Berlin and Humboldt-Universität zu Berlin, Charitéplatz 1, 10117 Berlin, Germany; georg.riepe@charite.de (G.R.); jonas.schunack@charite.de (J.S.); iwona.wallach@charite.de (I.W.); agustin.liotta@charite.de (A.L.); 3 Institute of Neurophysiology, Charité—University Medical Center Berlin, Corporate Member of Freie Universität Berlin and Humboldt-Universität zu Berlin, Charitéplatz 1, 10117 Berlin, Germany; joerg.roesner@charite.de; 4 Neuroscience Research Center, Charité—University Medical Center Berlin, Corporate Member of Freie Universität Berlin and Humboldt-Universität zu Berlin, Charitéplatz 1, 10117 Berlin, Germany; 5 Department of Experimental Neurology, Charité—University Medical Center Berlin, Corporate Member of Freie Universität Berlin and Humboldt-Universität zu Berlin, Charitéplatz 1, 10117 Berlin, Germany

**Keywords:** cerebral metabolic rate of oxygen, gamma oscillations, mitochondrial metabolism, neuronal hyperexcitability, S-ketamine

## Abstract

S-ketamine is increasingly used in anesthesia and neurocritical care and has been associated with anticonvulsant and potentially neuroprotective effects. Such neuroprotective effects have been observed particularly in states of severe hyperexcitability, including status epilepticus and spreading depolarizations, in which neuronal energy demand rises sharply and mitochondrial function may become compromised. However, the direct effects of S-ketamine on neuronal oxidative metabolism across different activity states remain incompletely understood. In acute hippocampal slices from 6–12-week-old male and female C57BL/6 mice, cerebral metabolic rate of oxygen (CMRO_2_) was quantified using depth-resolved tissue oxygen measurements under baseline conditions, during electrical stimulation, kainate-induced gamma oscillations, and Mg^2+^-free-induced epileptiform activity. Extracellular potassium dynamics and flavin adenine dinucleotide autofluorescence were recorded to assess neuronal excitability and mitochondrial redox state. S-ketamine was tested at concentrations of 100–1000 µM, and findings were integrated with computational modeling. At 100 µM, S-ketamine did not alter basal or stimulus-evoked CMRO_2_ or extracellular potassium handling. During gamma oscillations, S-ketamine modestly reduced CMRO_2_ and decreased oscillation frequency. Under Mg^2+^-free conditions, S-ketamine abolished seizure-like events and normalized CMRO_2_ to pre-Mg^2+^-free levels. Higher concentrations suppressed stimulus-evoked metabolism and attenuated mitochondrial redox responses, consistent with reduced neuronal activity rather than acute energy failure. These findings demonstrate an activity-dependent neurometabolic profile of S-ketamine characterized by preservation of oxidative metabolism under physiological conditions and reduced metabolic stress during pathological hyperexcitability.

## 1. Introduction

The brain is among the most energetically demanding organs, accounting for approximately 20% of total oxygen consumption at rest [1]. A substantial proportion of neuronal adenosine triphosphate (ATP) production is required to maintain transmembrane ion gradients and sustain synaptic transmission and network activity [2]. Therefore, neuronal function critically depends on a continuous supply of oxygen and glucose, rendering the brain particularly vulnerable to disturbances in energy metabolism [3]. Even modest impairment of oxidative metabolism can disrupt synaptic signaling, promote excitotoxic injury, and precipitate acute or delayed neurological dysfunction [2].

During pathological neuronal hyperexcitability, sustained discharges and the subsequent restoration of transmembrane ion gradients sharply increase ATP demand, which is met predominantly by mitochondrial oxidative phosphorylation, with an additional contribution from glycolysis [2]. In parallel, neuronal activity triggers neurovascular signaling involving neurotransmitter- and metabolite-dependent pathways, including nitric oxide, prostaglandins, adenosine, and K^+^, which increase local cerebral blood flow and thereby oxygen and substrate delivery [4]. Astrocyte–neuron metabolite exchange further supports the increased metabolic demand [2,3]. When the demand exceeds oxidative capacity, the resulting energy deficit impairs ion homeostasis and inhibitory interneuron function, leading to metabolic and excitatory disturbances that may reinforce one another [5,6].

General anesthetics profoundly alter neuronal activity, cerebral perfusion, and mitochondrial function, thereby directly affecting cerebral energy metabolism [7,8,9]. Reflecting these actions on neuronal excitability, many of these agents also exhibit intrinsic anticonvulsant properties, most commonly by potentiating γ-aminobutyric acid type A (GABA_A_) receptor-mediated inhibition (e.g., propofol, barbiturates, and volatile anesthetics) or by antagonizing excitatory N-methyl-D-aspartate (NMDA) receptors [10]. However, several anesthetics act as anticonvulsants only at specific doses [11], and some volatile agents such as sevoflurane can even become proconvulsant at high concentrations [12,13].

While suppression of neuronal activity and metabolic demand underlies the neuroprotective rationale for anesthesia in conditions such as traumatic brain injury or refractory status epilepticus, anesthetic-induced alterations in neurovascular coupling or mitochondrial respiration may conversely create states of energetic mismatch. Such metabolic vulnerability has been implicated in postoperative neurological complications, including delirium and long-term cognitive impairment [14,15]. A detailed understanding of how individual anesthetic agents modulate neuronal oxidative metabolism is therefore essential to distinguish metabolically adaptive from potentially harmful effects [9].

S-ketamine, the S-enantiomer of ketamine and a noncompetitive NMDA receptor antagonist, occupies a unique position among anesthetic agents and has gained increasing attention in neurocritical care and perioperative medicine [16,17,18]. Clinically, S-ketamine has been associated with reduced spreading depolarizations after subarachnoid hemorrhage [19,20], effective seizure suppression in refractory status epilepticus [21], and a lower incidence of postoperative delirium [22]. These observations have raised interest in its potential neuroprotective profile, particularly under conditions of pathological neuronal hyperactivity.

Experimental studies suggest that ketamine may modulate multiple cellular pathways implicated in neuroprotection, including excitotoxic signaling, oxidative stress, inflammation, and mitochondrial function [23,24,25]. Conversely, evidence of ketamine-induced mitochondrial dysfunction and neurotoxicity has also been reported, particularly at high concentrations or with prolonged exposure [26,27,28,29,30]. These seemingly contradictory findings underscore the context- and dose-dependent nature of ketamine’s effects. A systematic evaluation across a broad concentration range is therefore essential to delineate metabolically safe from potentially harmful effects.

Despite the extensive clinical and experimental literature, little is known about the acute effects of S-ketamine on neuronal oxidative metabolism itself. Most prior studies rely on indirect markers of mitochondrial function [29,30,31], histological outcomes [26], or long-term behavioral measures [32], whereas direct real-time measurements of cerebral oxygen consumption are lacking.

In previous work, we demonstrated that anesthetic agents differ fundamentally in their metabolic profiles: propofol can directly impair mitochondrial respiration, whereas volatile anesthetics primarily reduce energy demand through activity-dependent mechanisms [33,34,35]. Here, we aimed to define the neurometabolic profile of S-ketamine using an integrative ex vivo and in silico approach. Acute hippocampal slices allow direct measurement of neuronal oxidative metabolism under controlled oxygen and substrate supply, thereby minimizing confounding effects of systemic variables. In this study, we quantified cerebral metabolic rate of oxygen (CMRO_2_) across distinct activity states, including baseline conditions, physiological gamma oscillations, and epileptiform activity. We integrated these measurements with computational modeling of ATP dynamics and mitochondrial redox state. This approach allowed us to distinguish activity-dependent from potential direct metabolic effects of S-ketamine. We hypothesized that S-ketamine exerts a distinctive, activity-dependent metabolic profile that may underlie its clinical neuroprotective effects and distinguish it from other anesthetic agents.

## 2. Results

Data from both male and female animals were included in all experiments. Separate analyses for male and female animals revealed consistent directionality and comparable statistical significance of all main effects (Supplementary Tables S1–S6); data were therefore pooled for the main analyses.

### 2.1. S-Ketamine (100 µM) Does Not Affect Cerebral Metabolic Rate of Oxygen (CMRO_2_) or Potassium Handling in Naïve Slices

**Figure 1:**
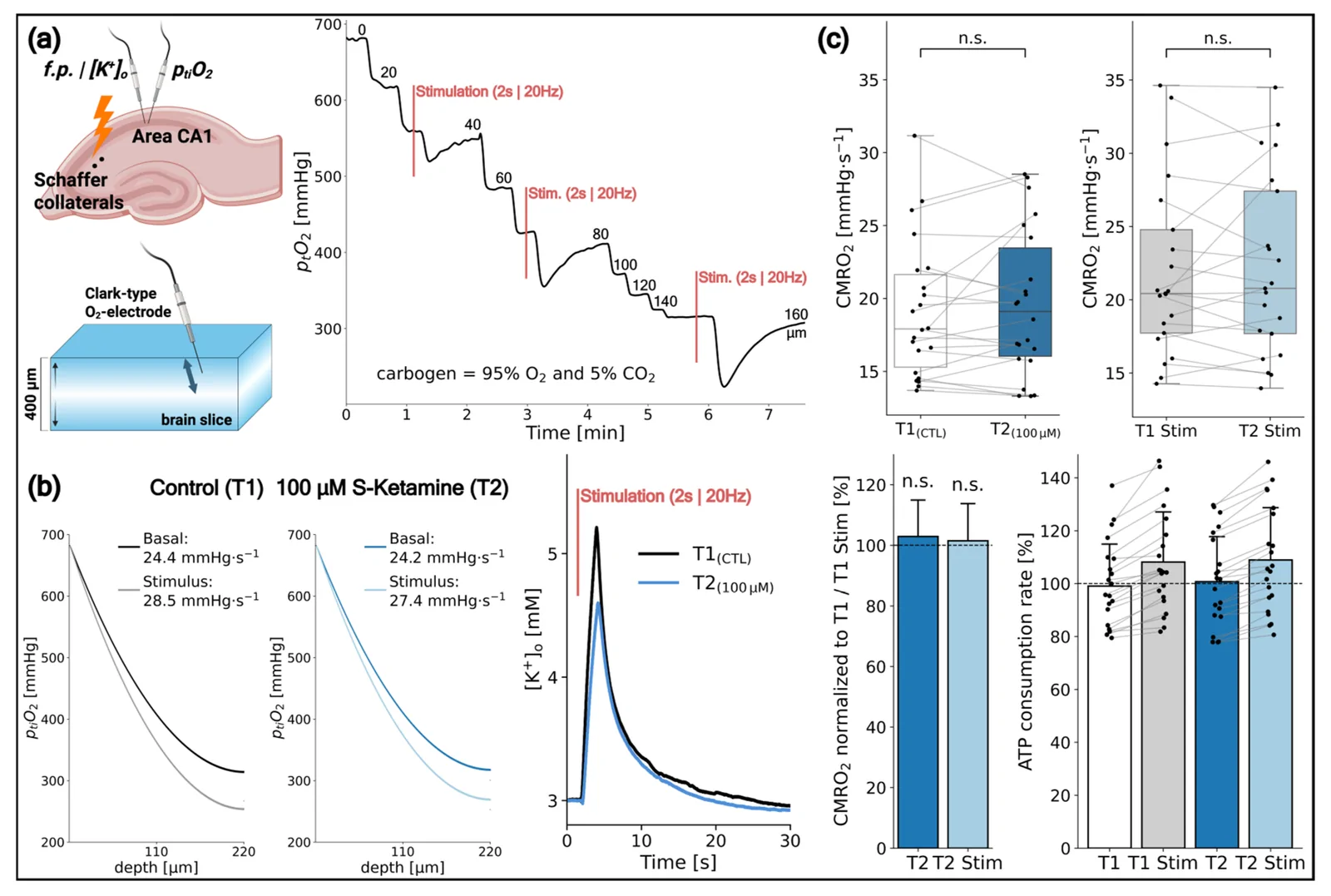
S-ketamine (100 µM) does not alter basal or stimulus-induced oxidative metabolism or potassium handling. (**a**) Schematic of electrode placement in hippocampal area CA1. Electrical stimulation was applied to the Schaffer collaterals. Representative partial tissue oxygen pressure (ptiO_2_) depth profiles obtained under interface conditions before and during electrical stimulation (20 Hz, 2 s) are shown. (**b**) Representative ptiO_2_ depth fits, calculated cerebral metabolic rate of oxygen (CMRO_2_), and stimulus-induced extracellular potassium ([K^+^]_o_) responses under control conditions and after application of 100 µM S-ketamine. (**c**) Absolute and normalized CMRO_2_ values and modeled relative adenosine triphosphate (ATP) consumption rates under basal conditions and during stimulation, before and after S-ketamine application. Data are shown as boxplots indicating median, interquartile range, and individual paired data points; bar graphs show mean + SD. *n* = 21–22 slices from three animals. Statistical analysis was performed using Wilcoxon signed-rank tests for paired comparisons of absolute and normalized CMRO_2_ values. No statistically significant differences were detected between control and S-ketamine conditions. n.s., not significant.

To assess whether S-ketamine alters neuronal energy metabolism at pharmacologically effective concentrations, we quantified CMRO_2_, modeled ATP consumption, and extracellular potassium ([K^+^]_o_) dynamics in hippocampal CA1 slices before and after application of 100 µM S-ketamine (Figure 1). Measurements were performed under baseline conditions and during brief electrical stimulation.

During baseline recordings, oxygen consumption remained stable at 17.90 mmHg·s^−1^ (14.80–21.97) before and 19.10 mmHg·s^−1^ (15.83–24.39) after S-ketamine application (*n* = 22; Wilcoxon signed-rank test, *p* = 0.548). Correspondingly, normalized CMRO_2_ changed non-significantly by 2.91% and modeled ATP consumption by 1.66% (Figure 1b,c). Following tetanic stimulation (20 Hz, 2 s), CMRO_2_ increased to 20.42 mmHg·s^−1^ (17.53–25.79) before and 20.77 mmHg·s^−1^ (16.95–27.79) after drug application (*n* = 21; Wilcoxon signed-rank test, *p* = 0.848). The normalized response showed no significant effect (+1.52%), and modeled ATP consumption remained unchanged (+0.75%; Figure 1b,c). Time-control experiments confirmed that basal and stimulus-induced CMRO_2_ remained stable over repeated measurements, excluding time-dependent drift (Supplementary Figure S2).

Stimulus-evoked [K^+^]_o_ transients were analyzed to assess neuronal excitability. Amplitudes were significantly reduced in both the untreated control group to 82.29% (74.23–92.06; *n* = 16; paired *t*-test, *p* < 0.001) and the S-ketamine group to 79.85% (73.32–97.75; *n* = 19; paired *t*-test, *p* = 0.015). There was no significant difference between groups (Welch *t*-test, *p* = 0.348), indicating a time-dependent decline due to inherent tissue dynamics during slice preparation rather than a drug-specific effect. Baseline [K^+^]_o_ levels and potassium clearance kinetics were likewise unaffected by S-ketamine (Supplementary Figure S3).

### 2.2. Higher S-Ketamine Concentrations Dose-Dependently Reduce CMRO_2_ and Excitability

**Figure 2:**
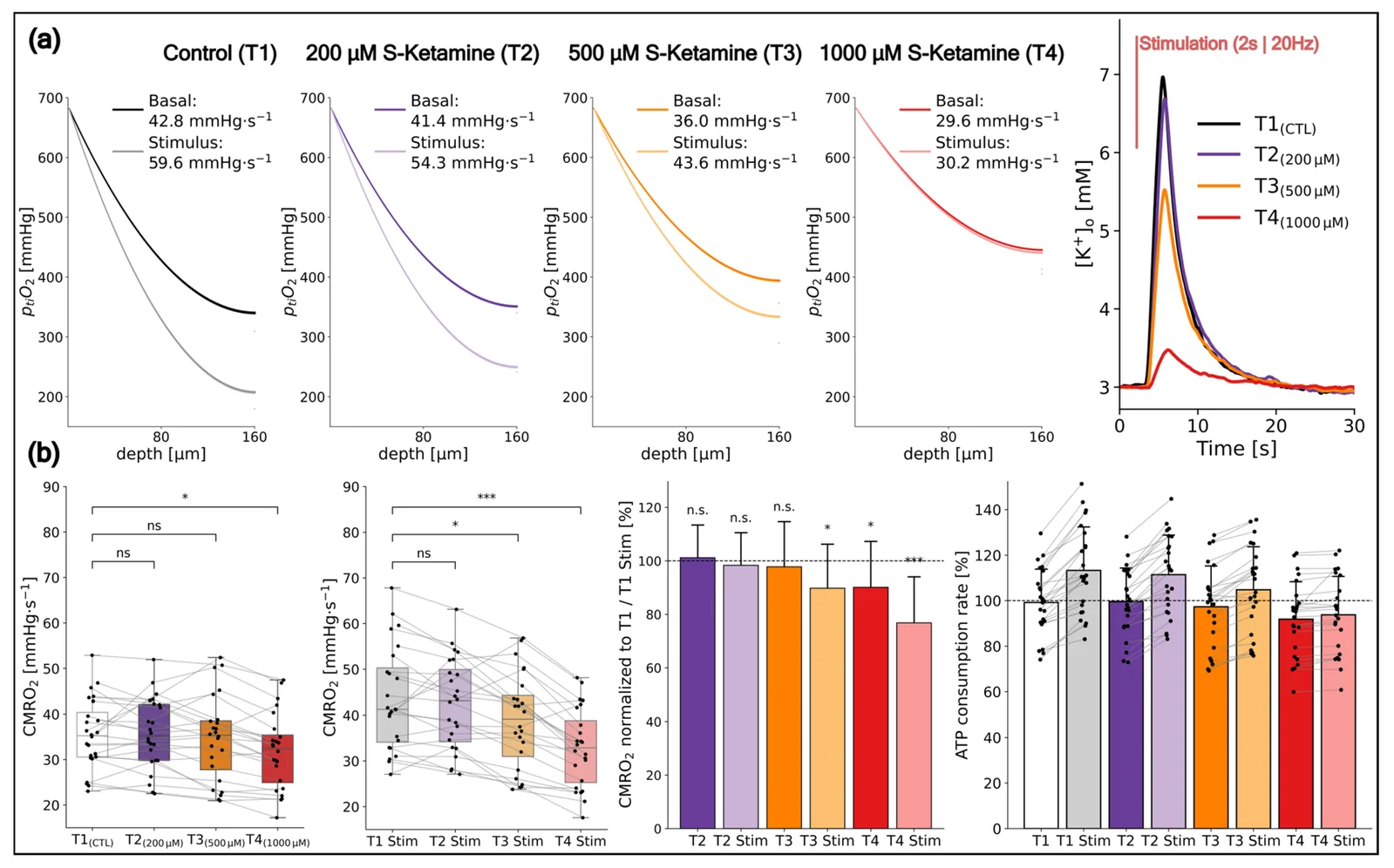
High concentrations of S-ketamine suppress stimulus-evoked CMRO_2_ and neuronal excitability. (**a**) Representative ptiO_2_ depth profiles, calculated CMRO_2_ values, and stimulus-induced [K^+^]_o_ responses under control conditions and after application of 200, 500, and 1000 µM S-ketamine. (**b**) Absolute and normalized CMRO_2_ values and modeled relative ATP consumption rates under basal conditions and during stimulation. Data are shown as boxplots indicating median, interquartile range, and individual paired data points; bar graphs show mean + SD. *n* = 24 slices from seven animals. Statistical analysis was performed using the Friedman test for repeated measures, followed by Bonferroni-corrected Wilcoxon signed-rank post hoc tests comparing each concentration with the control. Basal CMRO_2_ was significantly reduced at 1000 µM S-ketamine. Stimulus-induced CMRO_2_ was significantly reduced at 500 and 1000 µM. * *p* < 0.05, *** *p* < 0.001; n.s., not significant.

Since 100 µM S-ketamine did not measurably affect oxidative metabolism or potassium handling in naïve slices, we examined whether higher concentrations (200, 500, 1000 µM) induce concentration-dependent metabolic changes (Figure 2).

Under basal conditions, S-ketamine induced concentration-dependent reductions in CMRO_2_. CMRO_2_ measured 35.24 mmHg·s^−1^ (30.41–42.00) under control conditions, 35.19 (29.79–42.21) at 200 µM, 35.35 (26.12–38.69) at 500 µM, and 32.40 (24.03–36.29) at 1000 µM (*n* = 24; Friedman test, *p* < 0.001). Post hoc comparisons (Bonferroni-corrected Wilcoxon signed-rank tests) showed a significant reduction only at 1000 µM (*p* = 0.042; 200 µM *p* = 1.000; 500 µM *p* = 0.276). Normalized CMRO_2_ changed by +1.16% (200 µM), −2.25% (500 µM), and −9.91% (1000 µM). ATP consumption showed a comparable profile (+0.35%, −1.87%, −7.36%). Although these effects remained below 10%, the significant decrease at 1000 µM indicates a suppression of basal oxidative metabolism.

Following electrical activation, CMRO_2_ rose to 41.27 mmHg·s^−1^ (33.30–52.08) in controls, 43.16 (33.37–51.32) at 200 µM, 39.13 (28.99–45.68) at 500 µM, and 32.87 (24.45–40.71) at 1000 µM (*n* = 24; Friedman test, *p* < 0.001). Post hoc tests indicated significant reductions at 500 µM and 1000 µM (*p* = 0.013 and *p* < 0.001, respectively), but not at 200 µM (*p* = 0.390). Normalized CMRO_2_ changed by −1.65% (200 µM), −10.22% (500 µM), and −23.21% (1000 µM), and ATP consumption showed similar decreases (−1.88%, −8.52%, −19.50%). Oxygen consumption remained stable over time in untreated slices (Supplementary Figure S2).

Reductions in excitability paralleled the metabolic effects. Stimulation-evoked increases in [K^+^]_o_ were significantly reduced at 500 µM and 1000 µM (Supplementary Figure S3). At 500 µM, amplitude decreased to 50.67% (39.02–56.97) vs. 69.44% (54.98–77.28) in controls (*n* = 20 vs. 16; Bonferroni-corrected Student’s *t*-test, *p* = 0.003). At 1000 µM, it fell to 8.70% (7.17–17.61) vs. 57.13% (40.22–66.60) (Bonferroni-corrected Mann–Whitney U test, *p* < 0.001). No difference was observed at 200 µM (*p* = 1.000). Baseline [K^+^]_o_ and clearance kinetics were unaffected.

### 2.3. S-Ketamine (100 µM) Modestly Reduces CMRO_2_ During Gamma Oscillations and Slows Oscillation Frequency

**Figure 3:**
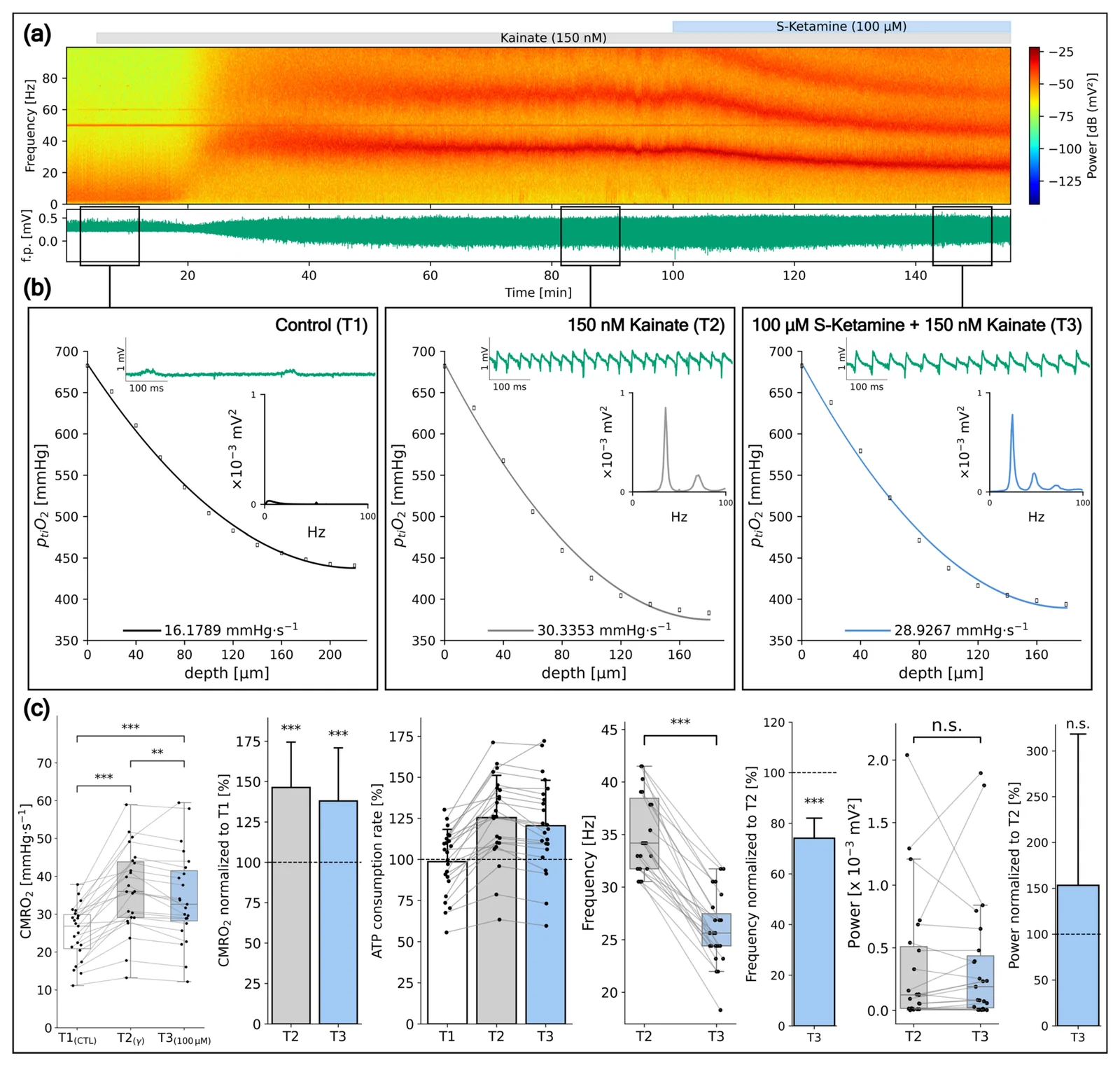
Effects of S-ketamine on kainate-induced gamma oscillations and associated metabolic demand. (**a**) Representative field potential recording and corresponding time–frequency spectrogram illustrating stable gamma oscillations induced by 150 nM kainate and their modulation by 100 µM S-ketamine. (**b**) Representative ptiO_2_ depth profiles and calculated CMRO_2_ under baseline conditions, during kainate-induced gamma oscillations, and during gamma oscillations in the presence of S-ketamine. (**c**) Absolute and normalized CMRO_2_ values, modeled relative ATP consumption rates, gamma frequency, and gamma power across conditions. Data are shown as boxplots indicating median, interquartile range, and individual paired data points; bar graphs show mean + SD. *n* = 23 slices from five animals. Statistical analysis was performed using the Friedman test for repeated measures followed by Bonferroni-corrected Wilcoxon signed-rank post hoc tests for absolute CMRO_2_ values. Normalized CMRO_2_ values were analyzed using Bonferroni-corrected paired *t*-tests against baseline. Gamma frequency was assessed using paired *t*-tests, and gamma power using Wilcoxon signed-rank tests. Kainate significantly increased CMRO_2_ compared with baseline. Addition of S-ketamine significantly reduced CMRO_2_ and gamma frequency, while gamma power remained unchanged. ** *p* < 0.01, *** *p* < 0.001; n.s., not significant.

Because basal neuronal activity in acute brain slices is relatively low and electrical stimulation induces only transient increases in energy demand, we investigated the effects of S-ketamine during sustained network activity by inducing gamma oscillations with bath application of kainate (150 nM) (Figure 3).

As expected, gamma induction markedly increased baseline oxygen consumption from 26.84 mmHg·s^−1^ (20.44–30.17) under control conditions to 35.99 mmHg·s^−1^ (29.08–44.14) during stable oscillations (*n* = 23; Bonferroni-corrected Wilcoxon signed-rank test, *p* < 0.001), corresponding to a 46.41% increase in normalized CMRO_2_ and a 26.83% rise in ATP consumption. Application of 100 µM S-ketamine caused a modest but significant reduction in oxygen consumption to 32.61 mmHg·s^−1^ (27.55–42.30; *p* = 0.001), while normalized CMRO_2_ and ATP consumption remained elevated by 37.98% and 21.88% relative to the baseline condition.

To assess how these metabolic changes relate to network dynamics, gamma oscillation frequency and power were analyzed. Gamma power changed non-significantly from 123 µV^2^ (15–541) to 189 µV^2^ (14–479) after S-ketamine application (*n* = 23; Wilcoxon signed-rank test, *p* = 0.616). Peak frequency showed a significant decrease from 34.18 Hz (31.74–39.06) to 25.63 Hz (24.41–28.08) after S-ketamine application (paired *t*-test, *p* < 0.001), corresponding to a normalized reduction of 25.90%.

### 2.4. S-Ketamine (100 µM) Abolishes Mg^2+^-Free-Induced Epileptiform Activity and Normalizes CMRO_2_ to Pre-Mg^2+^-Free Levels

**Figure 4:**
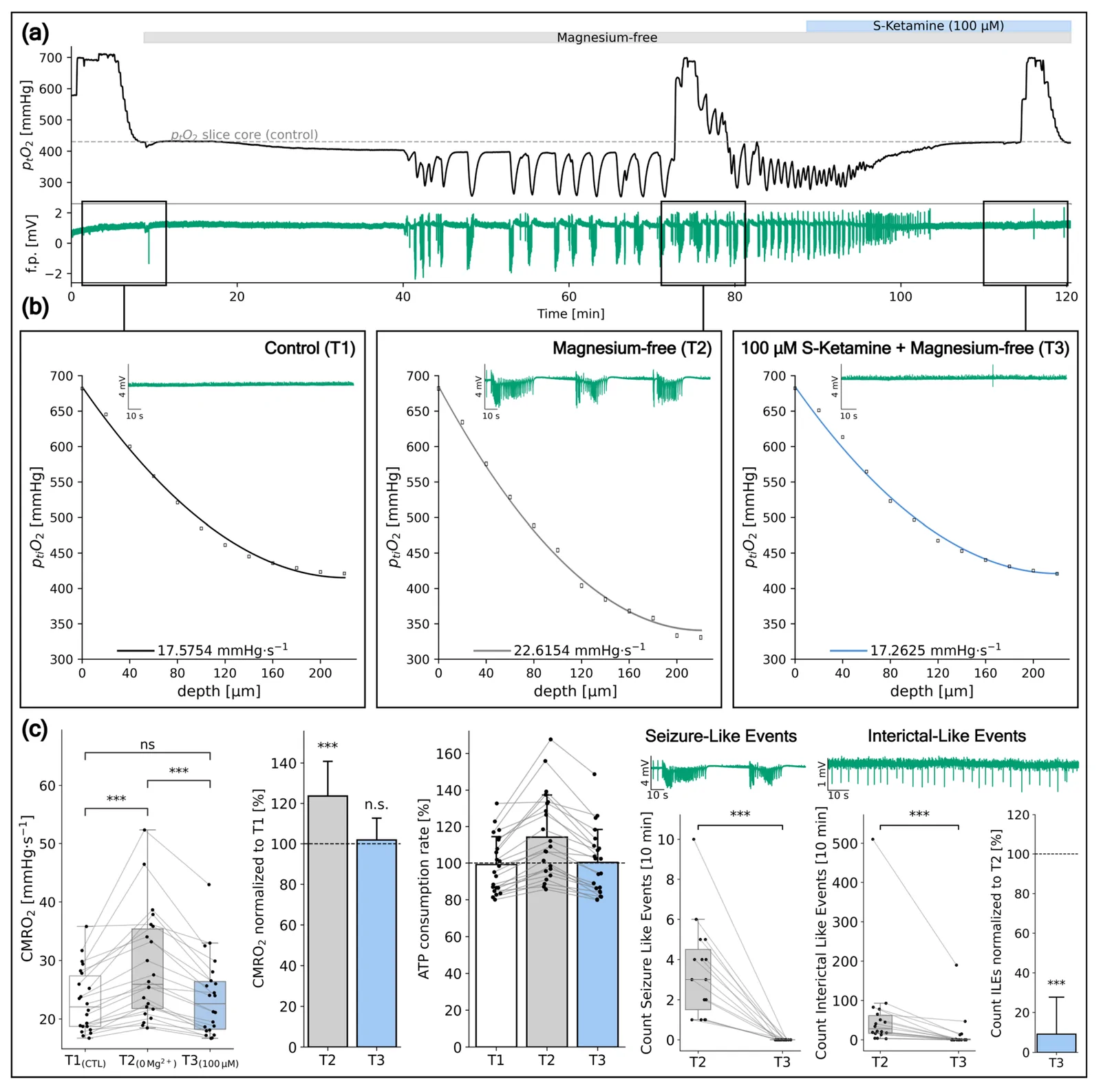
S-ketamine suppresses epileptiform activity and normalizes metabolic demand. (**a**) Representative field potential and ptiO_2_ recordings under control conditions, during Mg^2+^-free-induced epileptiform activity, and after addition of 100 µM S-ketamine. (**b**) Representative ptiO_2_ depth profiles and calculated CMRO_2_ values across experimental conditions. (**c**) Absolute and normalized CMRO_2_ values, modeled relative ATP consumption rates, number of seizure-like events (SLEs), and interictal-like events (ILEs). Data are shown as boxplots indicating median, interquartile range, and individual paired data points; bar graphs show mean + SD. *n* = 22 slices for metabolic measurements, *n* = 15 slices for SLE analysis, and *n* = 18 slices for ILE analysis, from 7–11 animals. Statistical analysis was performed using the Friedman test with Bonferroni-corrected Wilcoxon signed-rank post hoc tests for CMRO_2_. SLEs and ILEs were analyzed using Wilcoxon signed-rank tests. Mg^2+^-free conditions significantly increased CMRO_2_ and pathological event frequency. Application of S-ketamine abolished SLEs, markedly reduced ILEs, and normalized CMRO_2_ to pre-Mg^2+^-free levels. *** *p* < 0.001; n.s., not significant.

To investigate the effects of S-ketamine under pathological neuronal hyperexcitability and increased metabolic demand, spontaneous epileptiform activity was induced by perfusion with Mg^2+^-free artificial cerebrospinal fluid (aCSF; Figure 4).

Induction of epileptiform activity significantly increased baseline oxygen consumption, with CMRO_2_ rising from 22.05 mmHg·s^−1^ (18.58–27.91) to 25.91 mmHg·s^−1^ (21.48–35.90; *n* = 22; Bonferroni-corrected Wilcoxon signed-rank test, *p* < 0.001). Normalized CMRO_2_ increased by 23.67%, and modeled ATP consumption by 15.03%. Following S-ketamine application, CMRO_2_ returned to 22.61 mmHg·s^−1^ (18.07–26.91; *p* < 0.001 vs. Mg^2+^-free condition; *p* = 1.000 vs. control condition). Relative to the original control, normalized CMRO_2_ and ATP consumption remained slightly elevated (+1.84% and +1.17%, respectively).

S-ketamine also markedly reduced epileptiform activity. The number of seizure-like events (SLEs) decreased from 3.00 (1.00–5.00) to 0 (0–0) per 10 min (*n* = 15; Wilcoxon signed-rank test, *p* < 0.001). Interictal-like events (ILEs) declined from 28.00 (15.25–68.25) to 0 (0–4.75) per 10 min (*n* = 18; Wilcoxon signed-rank test, *p* < 0.001), corresponding to a normalized reduction of 90.92%.

### 2.5. High S-Ketamine Concentrations Attenuate Mitochondrial Redox Responses

**Figure 5:**
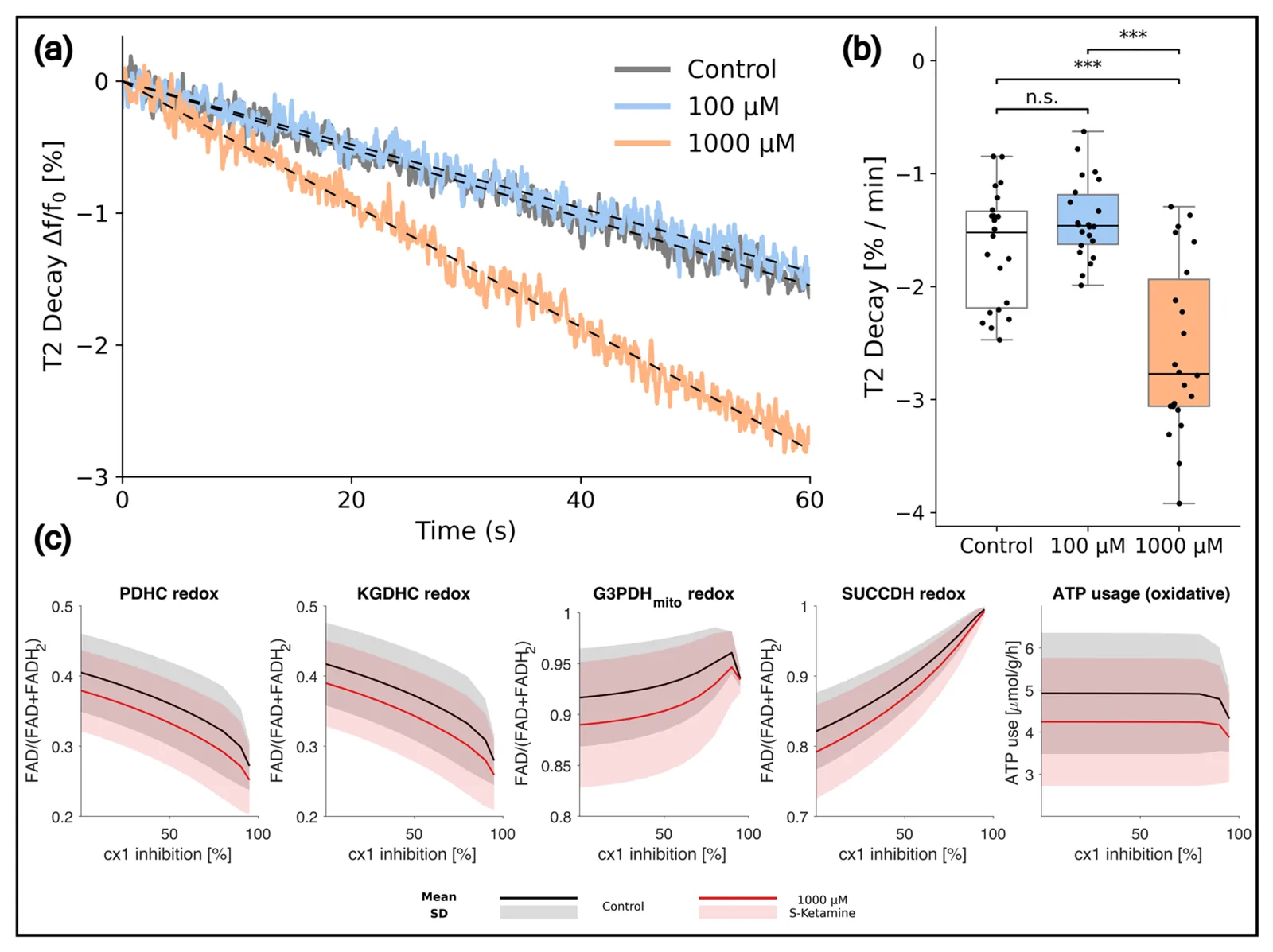
High S-ketamine concentrations alter baseline mitochondrial redox state. (**a**) Representative normalized flavin adenine dinucleotide (FAD) autofluorescence traces illustrating baseline decay under control conditions, 100 µM S-ketamine, and 1000 µM S-ketamine. (**b**) Quantification of FAD decay rates across conditions. (**c**) To mechanistically interpret the accelerated FAD decay observed at 1000 µM S-ketamine in the context of reported complex I inhibition by ketamine, we performed in silico simulations constrained by experimentally measured CMRO_2_ values. Basal ATP demand in the model was adjusted to reproduce CMRO_2_ under control conditions and after exposure to 1000 µM S-ketamine. Simulated steady-state FAD/(FAD+FADH_2_) ratios of pyruvate dehydrogenase complex (PDHC), α-ketoglutarate dehydrogenase complex (KGDHC), mitochondrial glycerol-3-phosphate dehydrogenase (G3PDH_mito_), and succinate dehydrogenase (SUCCDH), together with oxidative ATP usage, are shown as a function of complex I inhibition. Under both simulated conditions, mitochondrial ATP utilization remained stable across a wide range of simulated complex I activities, declining only when complex I capacity was reduced by more than 90%, indicating substantial respiratory reserve capacity in the preparation. The observed redox shift is most consistent with reduced metabolic demand secondary to suppressed neuronal activity. Partial complex I inhibition is not required to explain our findings, but it may contribute to the redox shift if the measured FAD signal is dominated by PDHC and KGDHC. Measured data are shown as boxplots indicating the median, interquartile range, and individual data points. *n* = 22 slices per group from 5–8 animals. Statistical analysis was performed using Welch ANOVA followed by Games–Howell post hoc tests. FAD decay was significantly accelerated at 1000 µM S-ketamine compared with control and 100 µM S-ketamine. No significant difference was detected between 100 µM S-ketamine and the control. *** *p* < 0.001; n.s., not significant.

Because high concentrations of S-ketamine reduced CMRO_2_ and attenuated stimulus-evoked metabolic responses, we examined whether these effects were associated with changes in mitochondrial redox state. We also tested whether 100 µM S-ketamine, which did not affect CMRO_2_, might nonetheless induce subtle mitochondrial alterations. To this end, we measured flavin adenine dinucleotide (FAD) autofluorescence as an indicator of the redox balance of FAD-containing mitochondrial enzymes and analyzed stimulus-induced FAD transients. Based on reports of ketamine-induced complex I inhibition, we complemented these measurements with simulations assessing partial complex I impairment (Figure 5).

Baseline FAD autofluorescence exhibited a gradual decay over time due to photobleaching (Figure 5a,b). The kinetics of this decay are modulated by shifts in the mitochondrial redox state [33,36]. Since illumination conditions were identical across groups, differences in decay rate can be attributed to drug-induced shifts in redox balance. In slices exposed to 1000 µM S-ketamine, the decay was significantly accelerated to 2.77%/min (1.81–3.07) compared with 1.52%/min (1.29–2.21) in control and 1.46%/min (1.14–1.65) in the 100 µM group (*n* = 22 per group; Games–Howell tests, all *p* < 0.001). No significant difference was detected at 100 µM vs. control (*p* = 0.165).

To determine whether the accelerated FAD decay at 1000 µM could reflect complex I inhibition, we performed simulations constrained by experimentally measured CMRO_2_ values (Figure 5c). Across both control and reduced-demand conditions, ATP utilization remained stable over a wide range of simulated complex I activities and declined only when activity was reduced by more than ~90%, indicating substantial respiratory reserve capacity.

**Figure 6:**
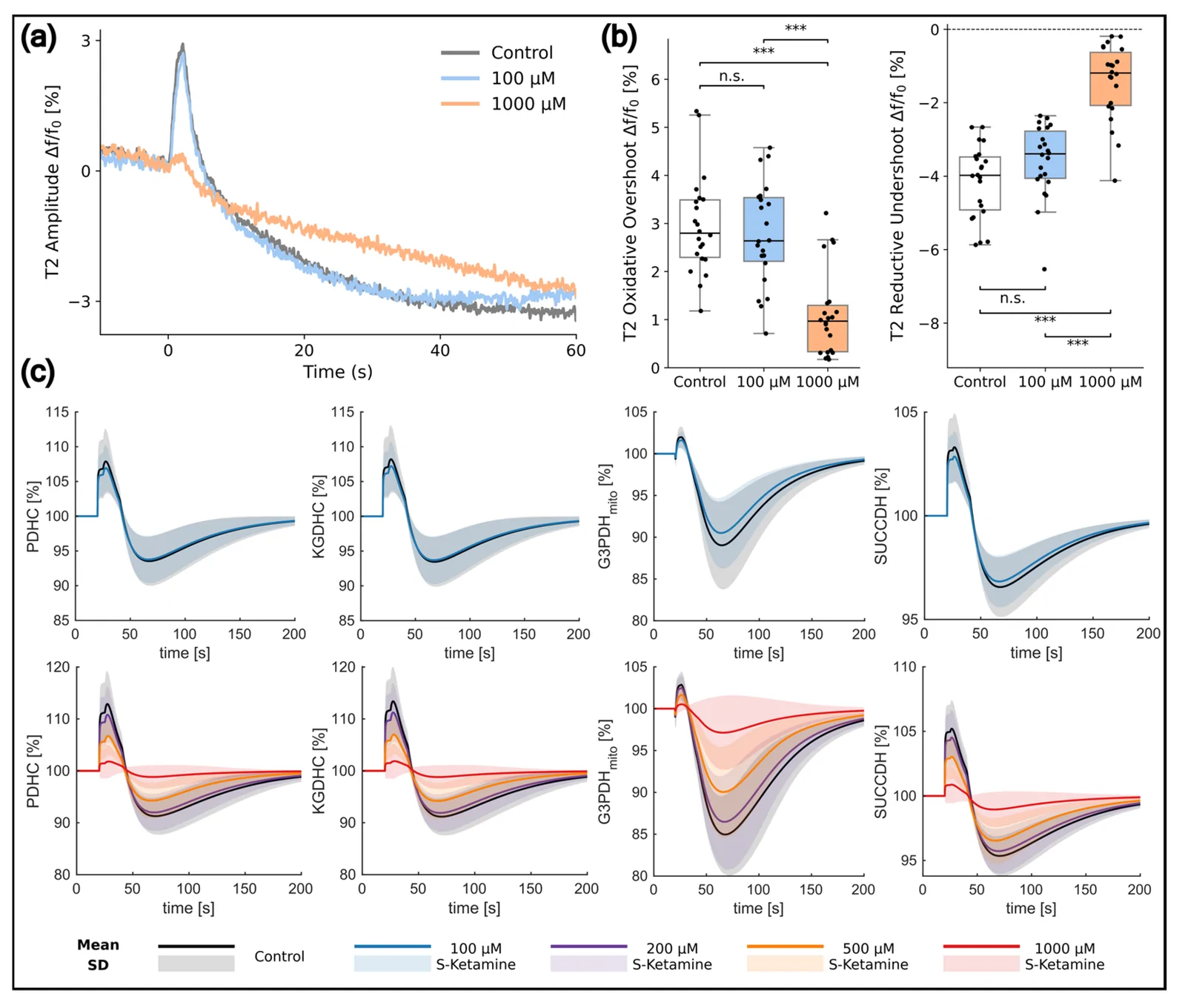
S-ketamine attenuates stimulus-induced mitochondrial redox responses. (**a**) Representative normalized stimulus-induced FAD transients under control conditions, 100 µM S-ketamine, and 1000 µM S-ketamine. (**b**) Quantification of oxidative overshoot and reductive undershoot amplitudes. (**c**) Simulated FAD redox responses of PDHC, KGDHC, SUCCDH, and G3PDH_mito_ during transient ATP demand increases, constrained by experimentally measured CMRO_2_ values. Across all simulated FAD-containing enzyme systems, including PDHC and KGDHC, the amplitudes of the oxidative overshoot and reductive undershoot decreased progressively as S-ketamine concentration increased. These simulated redox dynamics closely matched the experimentally observed attenuation of FAD transients at high S-ketamine concentrations. Measured data are shown as boxplots indicating the median, interquartile range, and individual data points. *n* = 22 slices per group from 5–8 animals. Statistical analysis was performed using the Kruskal–Wallis test followed by Bonferroni-corrected Dunn’s post hoc tests. Both oxidative overshoot and reductive undershoot were significantly reduced at 1000 µM S-ketamine compared with control and 100 µM S-ketamine. No significant differences were detected between 100 µM S-ketamine and the control. *** *p* < 0.001; n.s., not significant.

Stimulus-induced FAD transients exhibited the typical biphasic oxidative peak followed by a reductive undershoot (Figure 6a,b). At 1000 µM S-ketamine, both components were markedly reduced: the oxidative peak reached 0.97% (0.32–1.35) vs. 2.80% (2.26–3.51) in control and 2.64% (2.09–3.56) at 100 µM; the reductive undershoot decreased to 1.19% (0.53–2.11) vs. 3.98% (3.45–5.00) in control and 3.39% (2.70–4.10) at 100 µM (*n* = 22 per group; Bonferroni-corrected Dunn’s tests, all *p* < 0.001). At 100 µM, neither component differed significantly from the control (*p* = 1.000 and *p* = 0.459). Simulations reproduced the concentration-dependent attenuation of both transient components, supporting reduced mitochondrial responsiveness at high exposure (Figure 6c).

## 3. Discussion

In this study, we provide a comprehensive analysis of the acute neurometabolic effects of S-ketamine across distinct neuronal activity states using direct measurements of CMRO_2_, [K^+^]_o_ dynamics, mitochondrial redox readouts, and computational modeling. Our findings demonstrate that S-ketamine exerts strongly activity-, model-, and concentration-dependent effects on neuronal metabolism. Pharmacologically effective concentrations preserve basal and stimulus-evoked oxidative metabolism, mildly reduce CMRO_2_ during kainate-induced gamma oscillations, and normalize CMRO_2_ under Mg^2+^-free conditions. In contrast, higher concentrations suppress neuronal excitability and the associated metabolic demand, with no evidence of acute energy failure. These results refine current concepts of ketamine’s neurometabolic profile and have direct implications for its use in anesthesia and neurocritical care.

### 3.1. Considerations on S-Ketamine Concentrations in Acute Slice Experiments

A concentration of 100 µM S-ketamine was used to reliably engage NMDA receptor-dependent effects ex vivo, consistent with concentrations previously applied in brain-slice studies using racemic ketamine to suppress excitatory synaptic transmission, modulate or abolish spreading depolarizations, and attenuate network activity [37,38,39,40]. Although these concentrations exceed plasma levels typically achieved during clinical anesthesia [41], higher bath concentrations are required in acute slice preparations to compensate for diffusion barriers and tissue partitioning. Nevertheless, 100 µM is not intended to reproduce a specific anesthetic or neuroprotective dose. Its value lies instead in applying one defined, pharmacologically effective concentration across distinct activity states, thereby allowing general conclusions about the activity-dependence of its effects ex vivo.

In addition, substantially higher concentrations have been employed in mechanistic studies investigating mitochondrial function and cellular bioenergetics [29,31]. Such supratherapeutic concentrations are informative in metabolic studies, as they enable controlled induction of mitochondrial stress and increase sensitivity to bioenergetic vulnerabilities that are not detectable at lower concentrations.

### 3.2. Preservation of Oxidative Metabolism at Pharmacologically Effective Concentrations

S-ketamine (100 µM) did not alter basal CMRO_2_, stimulus-evoked increases in oxygen consumption, or ATP demand in naïve hippocampal slices. In our previous work, complete pharmacological blockade of postsynaptic glutamatergic (NMDA and α-amino-3-hydroxy-5-methyl-4-isoxazolepropionic acid) and GABAergic receptors reduced stimulus-induced oxygen transients by approximately 50% [42]. These findings suggest that partial NMDA receptor inhibition alone is insufficient to alter oxidative metabolism significantly and are particularly relevant given the high energetic requirements of neuronal signaling and the brain’s vulnerability to even modest disturbances in oxidative metabolism [1,2]. Preservation of [K^+^]_o_ transients further supports intact neuronal excitability and energy-dependent ion homeostasis, providing indirect but sensitive evidence that ATP availability and coupling between neuronal activity and metabolism remain intact.

At higher concentrations (200–1000 µM), S-ketamine progressively reduced activity-dependent CMRO_2_ and [K^+^]_o_ responses. The parallel decreases in stimulus-evoked CMRO_2_ and potassium transients indicate that these effects primarily reflect reduced neuronal excitability rather than impairment of mitochondrial oxidative capacity. Since NMDA receptor blockade is likely saturated within this range, additional targets are expected to contribute. S-ketamine has been shown to enhance GABA_A_ receptor-mediated inhibition [43,44] and to block voltage-gated sodium channels [45,46]. Both mechanisms are effective at concentrations comparable to those applied here and would reduce excitability. A significant reduction in basal CMRO_2_ was observed only at 1000 µM, indicating that oxidative metabolism is preserved over a wide range of concentrations.

Together, these findings provide no evidence for acute impairment of oxidative metabolism by S-ketamine at pharmacologically effective concentrations. Even at concentrations up to 500 µM, basal CMRO_2_ remained preserved, whereas activity-dependent reductions in CMRO_2_ were accompanied by decreased neuronal excitability. In contrast to anesthetic agents that directly depress oxidative metabolism independent of neuronal activity [33,47], these findings suggest that S-ketamine primarily modulates synaptic transmission and network dynamics while maintaining mitochondrial energy production.

### 3.3. Network-Dependent Metabolic Effects During Physiological Activity

To extend these observations beyond low baseline activity, we examined the effects of 100 µM S-ketamine during kainate-induced gamma oscillations, a model of sustained physiological network activity [48]. Gamma oscillations are generated by rhythmic perisomatic inhibition and are associated with high synaptic and metabolic activity [49,50].

As expected, gamma oscillations markedly increased CMRO_2_ and ATP consumption. During ongoing gamma oscillations, S-ketamine modestly reduced CMRO_2_ while metabolic demand remained elevated relative to baseline. Reduced CMRO_2_ was associated with decreased gamma frequency without a reduction in oscillation power, indicating altered temporal organization of network activity rather than suppression of synaptic transmission. Similar dissociations have been observed with NMDA antagonists, consistent with the modulation of interneuron–pyramidal cell interactions that shape oscillatory dynamics [51]. In contrast to the marked CMRO_2_ suppression observed with volatile anesthetics and propofol during comparable activity [33,34], these findings highlight distinct neurometabolic profiles among anesthetic agents.

### 3.4. Suppression of Pathological Hyperexcitability and Metabolic Normalization

In contrast to its limited effects under physiological conditions, S-ketamine exerted pronounced metabolic effects during Mg^2+^-free-induced epileptiform activity. Pathological hyperexcitability markedly increased CMRO_2_ and ATP demand, reflecting the energetic burden imposed by sustained seizure-like discharges. S-ketamine (100 µM) abolished SLEs, reduced interictal activity, and restored oxygen consumption to baseline levels recorded prior to Mg^2+^-free perfusion.

These findings provide direct metabolic evidence supporting the clinical efficacy of S-ketamine in refractory status epilepticus and related hyperexcitable states [19,20,21]. Notably, NMDA receptors are upregulated during status epilepticus, providing a mechanistic basis for the particular efficacy of NMDA antagonists [52]. Excessive neuronal firing during seizures can rapidly deplete cellular energy reserves, leading to ionic imbalance, excitotoxicity, and irreversible neuronal injury [5]. By suppressing pathological activity and thereby reducing metabolic demand, S-ketamine may help restore metabolic balance and limit secondary damage. Our data further suggest that this normalization arises from activity suppression rather than direct inhibition of mitochondrial function.

### 3.5. Mitochondrial Redox Dynamics at High Concentrations

Concerns regarding ketamine-induced mitochondrial toxicity largely stem from studies using high concentrations or prolonged exposure [27,28,29,53,54]. At 100 µM S-ketamine, baseline FAD autofluorescence decay and stimulus-induced redox transients were not altered, indicating preserved mitochondrial function. These findings are consistent with previous reports that ketamine exerts neuroprotective effects against oxidative and excitotoxic stress, including preservation of mitochondrial function [23,25].

Only at high concentrations (1000 µM) did we observe accelerated FAD decay and attenuation of stimulus-induced oxidative and reductive transients. Since FAD autofluorescence reflects the redox state of key mitochondrial enzymes, including pyruvate dehydrogenase complex (PDHC), α-ketoglutarate dehydrogenase complex (KGDHC), and succinate dehydrogenase (SUCCDH), it is sensitive to changes in flux rates and respiratory chain activity. The attenuated FAD transients at high S-ketamine concentrations therefore suggest reduced mitochondrial responsiveness to acute increases in energy demand. At the same time, the accelerated FAD decay indicates a shift toward a more reduced steady-state redox level.

### 3.6. Mechanistic Insight from Computational Modeling

To mechanistically interpret the redox changes, we complemented our experimental data with computational modeling constrained by measured CMRO_2_ values. The model, previously validated to reproduce neuronal energy metabolism and redox dynamics across activity states [35], demonstrated substantial respiratory reserve capacity. ATP utilization remained stable across a broad range of simulated complex I activities, declining markedly only when complex I capacity was reduced by more than 90%.

These findings suggest that partial inhibition of complex I, as reported at high ketamine concentrations [28,29,53,54], is unlikely to cause acute energy failure or to account for the decline in CMRO_2_ measured at 1000 µM. Instead, reduced metabolic demand secondary to suppressed neuronal activity likely explains the observed redox changes. The close correspondence between simulated and experimental FAD dynamics supports this interpretation. Partial complex I inhibition may nonetheless occur at this concentration but is not required to explain our findings.

### 3.7. Translational Implications for Anesthesia and Neurocritical Care

Consistent with S-ketamine’s mechanistic divergence from GABA_A_-potentiating anesthetics, our findings identify a distinctive neurometabolic profile of this noncompetitive NMDA receptor antagonist. Whereas propofol can directly impair mitochondrial function at high concentrations [33] and volatile anesthetics markedly suppress oxidative metabolism at clinically relevant doses [34,35], S-ketamine preserves mitochondrial oxidative capacity across a wide concentration range while selectively reducing demand during pathological hyperexcitability. This activity-dependent pattern may underlie its clinical utility in refractory seizures and acute brain injury [19,20,21].

Disturbed cerebral energy metabolism has long been proposed as a core mechanism of delirium [55]. In our previous work, we suggested that propofol-induced mitochondrial inhibition may create energy mismatch states after anesthesia, potentially contributing to postoperative cognitive dysfunction [33]. The preservation of oxidative metabolism in the present study is compatible with reports of a lower incidence of postoperative delirium or cognitive dysfunction following perioperative S-ketamine administration [22]. This may be particularly relevant in vulnerable patients with impaired cerebral perfusion or preexisting metabolic compromise. The metabolic contribution of anesthetics to the development of delirium remains to be tested in studies comparing S-ketamine-based anesthesia with regimens based on propofol or volatile agents under comparable analgesia and standardized delirium assessment, combined with intra- and postoperative measures of cerebral oxygen metabolism.

### 3.8. Limitations

Several limitations should be acknowledged. First, experiments were conducted in acute hippocampal slices lacking intact neurovascular coupling, which exhibit lower baseline metabolic demand than the in vivo brain. Absolute CMRO_2_ values, therefore, cannot be directly extrapolated to clinical conditions. However, within-slice comparisons across well-defined activity states provide high internal validity. Second, the concentrations applied ex vivo exceed reported clinical plasma concentrations and were administered only acutely. Differences between plasma and local brain exposure as well as between ex vivo and in vivo conditions limit direct quantitative translation, and clinical inferences will require validation at clinically relevant concentrations. Nevertheless, the absence of metabolic impairment at concentrations substantially above reported clinical plasma levels indicates a considerable safety margin with respect to the metabolic endpoints assessed here. Third, while CMRO_2_ and FAD autofluorescence provide information on mitochondrial respiration and redox state, reactive oxygen species (ROS) production and mitochondrial membrane potential were not assessed. Future studies incorporating ROS-sensitive probes and measurements of mitochondrial membrane potential could provide a more comprehensive assessment of mitochondrial function. The accompanying computational modeling likewise offers mechanistic insight but simplifies biological complexity. Fourth, epileptiform activity in the Mg^2+^-free model arises primarily from loss of the voltage-dependent Mg^2+^ block of NMDA receptors and is therefore not generalizable to NMDA receptor-independent forms of hyperexcitability. Finally, although sex-stratified analyses revealed consistent results (Supplementary Tables S1–S6), sample sizes were primarily designed to detect main effects rather than subtle sex-specific differences.

## 4. Materials and Methods

### 4.1. Animals, Slice Preparation, and Maintenance

All experimental procedures were approved by the responsible German authorities (Landesamt für Gesundheit und Soziales, Berlin, Germany) under approval number T-CH 0039/21 in January 2021 and conducted in accordance with the ARRIVE 2.0 guidelines [56], the Declaration of Helsinki, and institutional animal welfare policies (Charité—University Medical Center Berlin). Animals were group-housed (≥2 per cage) under a 12:12 h light–dark cycle with ad libitum access to food and water.

A total of 175 acute hippocampal slices were obtained from 41 wild-type C57BL/6 mice (20 male, 21 female) aged 6–12 weeks. A detailed sex-stratified summary of all experimental data is provided in the Supplementary Material. Slice preparation followed protocols originally established for rat brain tissue, with minor modifications [57]. Mice were anesthetized with 3% isoflurane vaporized in medical oxygen (CONOXIA; Linde, Pullach, Germany) to avoid tissue hypoxia, using a calibrated vaporizer (Dräger, Lübeck, Germany; flow rate: 2 L/min) connected to a custom-built induction chamber. Following decapitation, brains were rapidly extracted and horizontally sectioned into 400-µm-thick slices using a vibratome (VT1200S; Leica, Wetzlar, Germany). Slices were transferred to an interface-type holding chamber and perfused with aCSF equilibrated with carbogen (95% O_2_ and 5% CO_2_, continuous gas flow of 1 L/min). The composition of aCSF was (in mM): 129 NaCl, 21 NaHCO_3_, 10 glucose, 3 KCl, 1.25 NaH_2_PO_4_, 1.6 CaCl_2_, and 1.8 MgCl_2_; pH 7.35–7.45; osmolarity 295–305 mOsmol/L. Slices were allowed to recover for at least 2 h before recordings. aCSF flow was 2 mL/min in the interface and 10 mL/min in the submerged condition. The chambers were maintained at 36 °C.

### 4.2. Electrophysiology, Partial Tissue Oxygen Pressure (ptiO_2_) Recordings, and Fluorescence Imaging

Electrophysiology and partial tissue oxygen pressure (ptiO_2_) recordings were performed in the stratum pyramidale of hippocampal area CA1 under interface conditions. CA1 was chosen as the target of the stimulated Schaffer collaterals from CA3 and a principal output region of the hippocampus, projecting to the subiculum and entorhinal cortex [58]. Its dense laminar organization allows reproducible depth-resolved ptiO_2_ and FAD measurements. CA1 is a prominent site of seizure expression, integrating both Schaffer collateral input from CA3 and direct entorhinal projections [59].

Where applicable, electrical stimulation was delivered via a bipolar platinum electrode (20 µm diameter) placed in the stratum radiatum at the CA3–CA1 border to activate Schaffer collaterals originating from CA3. This paradigm reliably probes activity-dependent metabolic demand in hippocampal networks [33,35,42]. Simultaneously, field potentials, [K^+^]_o_, and ptiO_2_ were recorded. ptiO_2_ was measured using Clark-type oxygen microelectrodes (tip diameter: 10 µm; Unisense, Aarhus, Denmark). Electrodes were polarized for at least 2 h prior to use and calibrated before each experiment by a two-point calibration in aCSF equilibrated with atmospheric air (21% O_2_) and carbogen (95% O_2_/5% CO_2_). [K^+^]_o_ was assessed using double-barreled ion-selective microelectrodes constructed and calibrated according to Angamo et al. [60], using Potassium Ionophore I 60031 (Fluka, Buchs, Switzerland). For depth profiling, the oxygen sensor was advanced vertically in 20-µm steps through the slice using a micromanipulator (Narishige, Setagaya, Japan) until a local minimum in ptiO_2_ was reached, as previously described [61]. This procedure was repeated for each recording condition. The ion-selective microelectrode was positioned in proximity to the oxygen sensor and remained stationary at a depth of 50–100 µm throughout the entire recording. Activity-dependent changes in ptiO_2_ and [K^+^]_o_ were elicited by 2 s, 20 Hz stimulus trains (pulse width: 100 µs; inter-pulse interval: 50 ms; 40 pulses) delivered through the bipolar electrode. Pulse generation was controlled using a stimulator (Master-8; A.M.P.I., Jerusalem, Israel). When applicable, stimulation was repeated at standardized different slice depths (40 µm, 80 µm, and center) to assess spatial profiles. Activity-dependent and basal CMRO_2_ were measured during the same experiment.

FAD autofluorescence imaging was performed under submerged conditions using a custom-built setup. A 20× water-immersion objective (numerical aperture 0.5; Zeiss, Oberkochen, Germany) was focused on the stratum pyramidale of area CA1 under transmitted-light guidance to obtain autofluorescence from neuronal somata. Excitation was provided by a 460 nm LED (Lumen, Prior Scientific Ltd., Cambridge, UK) and spectrally filtered using a blue-bandpass fluorescence filter set (excitation BP470–495 nm, dichroic mirror DM505, emission BP510–550 nm; Olympus, Tokyo, Japan). Emitted fluorescence was detected using a photomultiplier tube (PMT, Seefelder Messtechnik, Oberhaching, Germany), providing a spatially integrated voltage signal. An LED intensity of 18% yielded a favorable balance between signal intensity and FAD photobleaching.

FAD autofluorescence declines over time due to photodecomposition of the flavin chromophore [62,63]. Because this decay is non-linear, direct comparison of different time points within the same slice is not feasible. To account for this, we designed three experimental groups, each with two recording time points. In all groups, the first measurement was performed under control conditions without drug application. The second measurement was then obtained after 10 min either (i) again under control conditions, (ii) after wash-in of 100 µM S-ketamine, or (iii) after wash-in of 1000 µM S-ketamine (Supplementary Figure S1). FAD decay rates were quantified as the slope of baseline fluorescence over a defined pre-stimulation interval. Stimulus-induced FAD transients were quantified as the amplitudes of the oxidative overshoot and reductive undershoot relative to baseline, reflecting mitochondrial redox responsiveness [33].

### 4.3. S-Ketamine Application and Induction of Gamma Oscillations and Spontaneous Pathological Activity

S-ketamine (Ketanest S, 25 mg/mL, 2 mL ampoules; Pfizer, Berlin, Germany) was applied at concentrations of 100 µM, 200 µM, 500 µM, and 1000 µM, dissolved in aCSF and continuously perfused into the recording chamber for at least 30 min under interface conditions (2 mL/min) and 10 min under submerged conditions (10 mL/min). The concentration range was selected based on prior experimental and clinical literature [41,43,45]. S-ketamine at 100 µM was used to reliably elicit NMDA receptor-dependent effects ex vivo [38,39], and higher concentrations (200–1000 µM) to resolve concentration-dependent effects and potential mitochondrial stress not detectable at 100 µM. Further considerations regarding the applied concentrations are provided in the Discussion (Considerations on S-ketamine concentrations in acute slice experiments).

Consistent with the literature [64], gamma oscillations were induced by bath application of 150 nM kainate (naturally derived; Tocris Bioscience, Bristol, UK) for 60 ± 5 min until stable gamma activity was observed in the field potential and the corresponding power spectrum. SLEs and ILEs were initiated by perfusion with Mg^2+^-free aCSF as previously described [57].

To exclude time-dependent drift as a confounder, time-control experiments without drug application were performed under identical recording conditions (Supplementary Figures S1–S3).

### 4.4. Data Acquisition and Analysis

The study used a within-slice paired design, with each slice serving as its own control across sequential conditions. The experimental unit (n) was defined as an individual slice; slices were obtained from multiple animals, as specified in the figure captions. For the FAD experiments (between-slice design due to photobleaching), slices were assigned to groups without formal randomization, with equivalent pre-intervention baselines (Supplementary Figure S1). Sample sizes were chosen based on prior studies from our group using the same experimental approach [34,35,57]. No formal a priori power calculation was performed. All recordings in which the targeted signal could be acquired were analyzed, and no data points were excluded post hoc. Recordings irreversibly compromised during acquisition (e.g., loss of evoked activity, electrode failure, or lasting signal alteration from mechanical disturbance such as a condensation droplet) were not entered into the dataset. Investigators were not blinded because drug application and activity paradigms required real-time adjustment of perfusion and recording parameters; outcome quantification used predefined, standardized analysis routines, including automated MATLAB R2023b (MathWorks Inc., Natick, MA, USA) scripts, to limit observer bias.

Analog signals were digitized using a Power CED1401 interface and acquired with Spike2 software version 6.09 (both from Cambridge Electronic Design, Cambridge, UK). Data analysis and statistical analyses were performed using Spike2, Microsoft Excel (Microsoft, Redmond, WA, USA), MATLAB, Python 3.9 (Python Software Foundation), and SPSS 29 (IBM, Armonk, NY, USA). Summary data are presented as medians with interquartile ranges (25th–75th percentiles) or as mean percentage changes relative to baseline calculated within each slice. Box plots display the median, interquartile range, and individual data points connected by lines across conditions. Bar charts display mean + SD. In normalized panels, asterisks denote comparisons against the reference condition indicated by the dashed line. FAD autofluorescence traces were smoothed with a 0.2 s moving average and analyzed using custom MATLAB scripts. No background subtraction was applied. Fluorescence changes following stimulation were expressed as ΔF/F_0_, where F_0_ corresponds to the fluorescence signal at the time point of stimulation onset (t = 0 s). To quantify fluorescence decay, a linear fit was applied to the 60 s baseline period preceding the stimulus (−60 s to 0 s). The resulting slope was normalized to the signal value at t = 0 s and expressed as percent change per minute. SLEs and ILEs were identified in the [K^+^]_o_ channel or the field potential channel and quantified within 10 min intervals corresponding to the respective recording sections. Power spectra of gamma oscillations were calculated from 5 min recordings per condition using fast Fourier transformation with a 4096-point Hanning window in Spike2.

For statistical analyses, distributional assumptions were assessed using the Shapiro–Wilk test for normality, Levene’s test (mean-based) for homogeneity of variances, and Mauchly’s test for sphericity. Paired data were analyzed using paired *t*-tests or Wilcoxon signed-rank tests, depending on normality. Independent two-group comparisons were performed using unpaired *t*-tests (with Welch correction when variances were unequal) or the Mann–Whitney U test for nonparametric data. When multiple independent two-group comparisons were performed, *p*-values were Bonferroni-corrected for the number of comparisons. Repeated-measures data with more than two time points were analyzed using repeated-measures ANOVA (with Greenhouse–Geisser correction when sphericity was violated) or the Friedman test for nonparametric data. Comparisons of more than two independent groups were evaluated using one-way ANOVA (Welch ANOVA when variances were unequal) or the Kruskal–Wallis test when assumptions for parametric testing were not met. Post hoc analyses were performed using Bonferroni-corrected paired *t*-tests after repeated-measures ANOVA, Tukey’s HSD after one-way ANOVA, Games–Howell after Welch ANOVA, and Bonferroni-corrected pairwise Wilcoxon signed-rank and Dunn’s tests following the Friedman and Kruskal–Wallis tests, respectively. Percent changes (normalized to the respective control condition within each experiment) were calculated to ensure comparability. Statistical significance was defined as *p* < 0.05.

### 4.5. Calculation of Cerebral Metabolic Rate of Oxygen

As described previously, CMRO_2_ was derived from ptiO_2_ depth profiles [61]. Basal CMRO_2_ was determined from the resting ptiO_2_ values recorded at each depth, whereas activity-dependent CMRO_2_ was calculated from the transient minimum ptiO_2_ after stimulation. Briefly, oxygen transport within the slice was modeled using a reaction–diffusion framework that accounts for both diffusive oxygen transport and metabolic oxygen consumption. The tissue was discretized into layers of equal thickness (1 µm). Oxygen diffusion between adjacent layers was governed by Fick’s law, using a diffusion coefficient of 1.6 × 10^3^ µm^2^/s. Oxygen consumption within each layer followed Michaelis–Menten kinetics with a K_m_ of 3 mmHg [65]. CMRO_2_ was assumed to be spatially homogeneous across the slice and was treated as a free parameter optimized to fit the experimental data. Boundary conditions were defined by fixing the ptiO_2_ at the slice surface to the supplied oxygen level, while imposing zero diffusive flux at the location of the minimum ptiO_2_.

### 4.6. Calculations of Flavin Adenine Dinucleotide (FAD) Transients and Adenosine Triphosphate (ATP) Consumption Rates

Because changes in FAD fluorescence originate from the activity of the PDHC, KGDHC, glycerol-3-phosphate dehydrogenase, and SUCCDH, fluorometric FAD measurements provide insight into the mitochondrial redox state [36,66]. Based on the calculated CMRO_2_ values, we used an established model of neuronal energy metabolism [36] to simulate FAD transients and ATP consumption rates. These modeled values are reported descriptively without separate statistical testing. Variations in basal CMRO_2_ reflect corresponding changes in ATP consumption, as increased ATP demand lowers intracellular ATP levels, thereby activating glycolysis, the citric acid cycle, and the respiratory chain, which in turn elevate CMRO_2_.

Alterations in basal ATP consumption resulting from S-ketamine, kainate, and/or Mg^2+^-free conditions were simulated by adjusting the ATP demand to reproduce the experimentally observed CMRO_2_. In experiments involving electrical stimulation, we also simulated the time-dependent metabolic response to a brief stimulus-induced increase in ATP demand, accompanied by a cytosolic Ca^2+^ transient, superimposed on the S-ketamine-dependent changes in the metabolic resting state. The temporal profile of the energetic load—that is, the stimulation-induced increase in ATP demand—was represented by a rectangular activation function corresponding to a short period of elevated metabolic demand matching the duration of stimulation. The associated cytosolic Ca^2+^ transient was modeled as a rapid increase reflecting stimulus-induced Ca^2+^ influx, followed by a slowly decaying phase representing Ca^2+^ extrusion from the cytosol.

Stimulus strength was determined from the calculated CMRO_2_ values under control conditions and after S-ketamine application. Mitochondria rapidly take up cytosolic Ca^2+^ via the Ca^2+^ uniporter, where it is initially buffered by Ca^2+^-binding proteins and subsequently released into the mitochondrial matrix. There, Ca^2+^ activates mitochondrial PDHC, isocitrate dehydrogenase, and KGDHC. Concurrently, increased ATP demand reduces cytosolic ATP levels, thereby stimulating glycolysis, cellular shuttle systems, and mitochondrial activity. The resulting changes in the redox state of protein-bound FAD were compared with experimentally measured FAD fluorescence signals to validate the model. Motivated by prior reports of ketamine-induced complex I inhibition [28,29,53,54], the FAD redox state was simulated under conditions of respiratory chain complex I inhibition by successively reducing its maximal activity (V_max_) from 100% to 5%, corresponding to inhibition levels from 0% to 95%.

## 5. Conclusions

Under ex vivo conditions, S-ketamine preserves neuronal oxidative metabolism at pharmacologically effective concentrations while selectively reducing metabolic demand during pathological neuronal hyperactivity or at high exposure levels. By maintaining mitochondrial function and energy homeostasis under physiological conditions and restoring metabolic balance during excessive activity, S-ketamine exhibits a neurometabolic profile potentially favorable for anesthesia and neurocritical care. These findings provide mechanistic support for its metabolic safety and potential neuroprotective effects and highlight the importance of activity-dependent evaluation of anesthetic actions on brain metabolism.

## Data Availability

The original contributions presented in this study are included in the article/Supplementary Material. Further inquiries can be directed to the corresponding author.

## Supplementary Materials

The following supporting information can be downloaded at https://www.mdpi.com/article/10.3390/ijms27188220/s1.

## Abbreviations

The following abbreviations are used in this manuscript:

aCSF	Artificial cerebrospinal fluid
ATP	Adenosine triphosphate
CMRO_2_	Cerebral metabolic rate of oxygen
FAD	Flavin adenine dinucleotide
G3PDH_mito_	Mitochondrial glycerol-3-phosphate dehydrogenase
GABA_A_	γ-Aminobutyric acid type A
ILEs	Interictal-like events
[K^+^]_o_	Extracellular potassium (concentration)
KGDHC	α-Ketoglutarate dehydrogenase complex
NMDA	*N*-methyl-D-aspartate
PDHC	Pyruvate dehydrogenase complex
ptiO_2_	Partial tissue oxygen pressure
ROS	Reactive oxygen species
SLEs	Seizure-like events
SUCCDH	Succinate dehydrogenase
