# Supplementary material for: S-Ketamine Preserves Oxidative Metabolism and Reduces Metabolic Stress During Neuronal Hyperexcitability
Source: Int J Mol Sci. 2026 Sep 15;27(18):8220. doi: 10.3390/ijms27188220 (PMC13607510; doi:10.3390/ijms27188220)
Supplement: Supplementary file 1 [file ijms-27-08220-s001.zip › ijms-4481137-supplementary.pdf]

Supplementary Material

Table of Contents

1. Supplementary Figures **........ 2**

Supplementary Figure S1........ 2

Supplementary Figure S2........ 3

Supplementary Figure S3........ 4

2. Supplementary Results Tables **........ 5**

Supplementary Table S1. 5

Supplementary Table S2. 6

Supplementary Table S3. 9

Supplementary Table S4. 10

Supplementary Table S5. 11

Supplementary Table S6. 12

1. Supplementary Figures

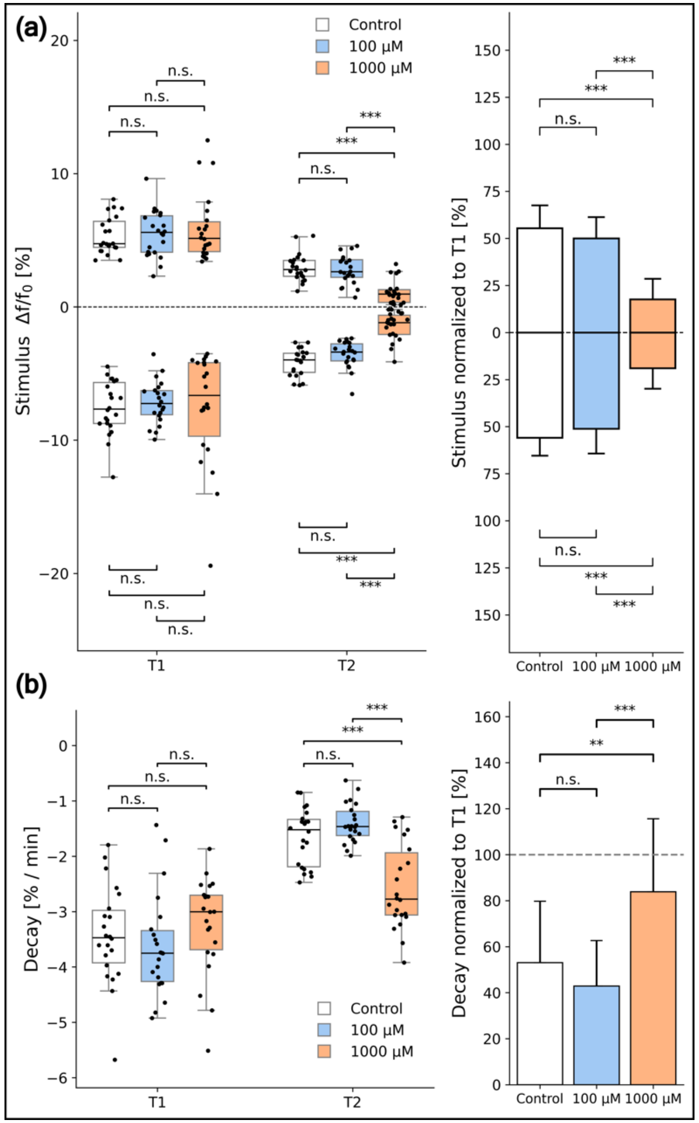

Supplementary Figure S1. FAD transients and decay at T1 (baseline) and after application of 100 µM S-ketamine (blue), 1000 µM S-ketamine (red), or no pharmacological intervention (white) at T2. **(a)** Absolute and normalized oxidative overshoot (top) and reductive undershoot (bottom). At T1, no significant differences were detected between groups ($p = 0.979$ and $p = 0.482$; Kruskal–Wallis test, respectively). At T2, application of 1000 µM S-ketamine significantly reduced both the oxidative overshoot and the reductive undershoot compared to the control condition and 100 µM S-ketamine. No significant differences were observed between 100 µM S-ketamine and control. Normalized FAD transients to T1 showed an identical pattern. **(b)** Absolute and normalized FAD decay. At T1, no significant differences were detected between groups ($p = 0.353$, one-way ANOVA). At T2, perfusion with 1000 µM S-ketamine significantly accelerated FAD photobleaching compared to control and 100 µM S-ketamine, indicating a shift toward a more reduced steady-state redox level. No significant differences were detected between 100 µM S-ketamine and control. Normalized decay to T1 depicted the same pattern. Boxplots show the median, interquartile range (IQR), and individual data points. Bar charts display mean + SD. $n = 22$ slices per group from 5–8 animals. Statistical comparisons: One-way ANOVA, Welch ANOVA, or Kruskal–Wallis test, followed by Tukey's HSD, Games–Howell, or Bonferroni-corrected Dunn's post hoc tests, depending on data distribution and variance homogeneity. ** $p < 0.01$, *** $p < 0.001$; n.s., not significant.

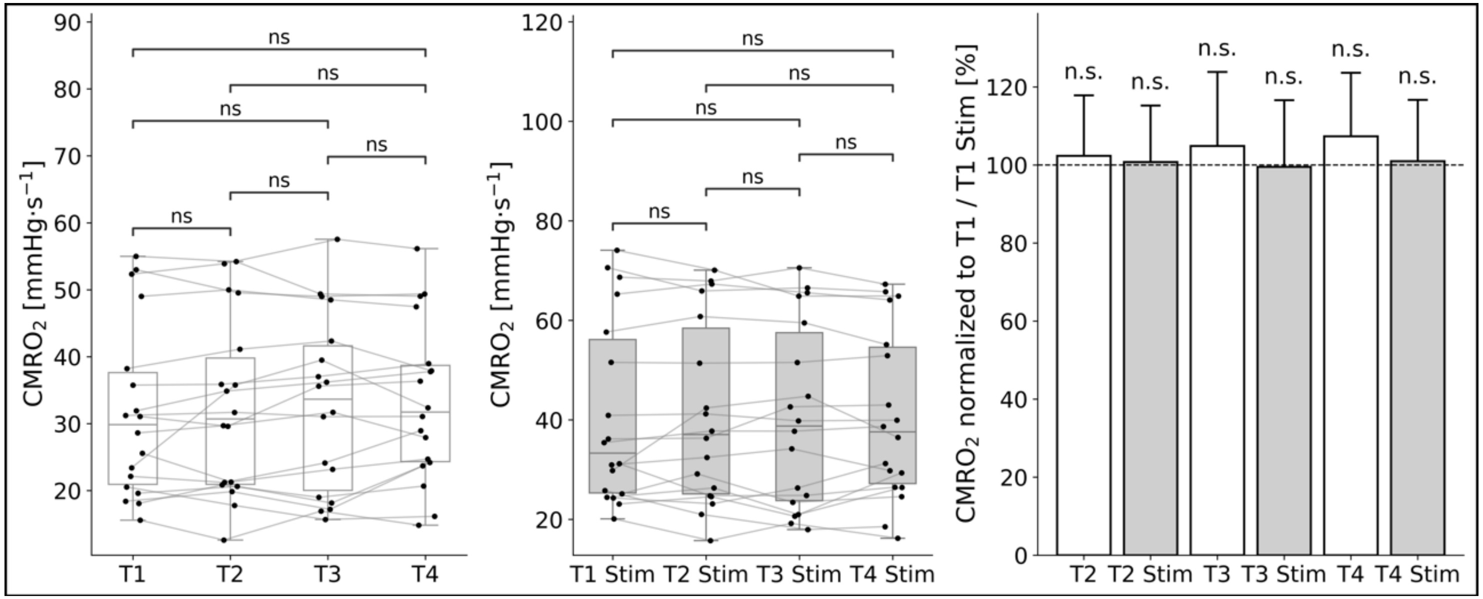

Supplementary Figure S2. Basal and stimulus-induced $CMRO_2$ in brain slices without pharmacological intervention over time. The minimum interval between measurement points was 40 minutes. Basal and stimulus-induced $CMRO_2$ showed no significant changes over time ($p = 0.926$, Friedman test, and $p = 0.974$, repeated-measures ANOVA, respectively). Normalized $CMRO_2$ values revealed a similar pattern. Boxplots show the median, interquartile range (IQR), and individual data points connected by lines across conditions. Bar charts display mean + SD. n = 18 slices from 6 animals. Statistical comparisons: Friedman tests followed by Bonferroni-corrected Wilcoxon signed-rank tests for absolute basal $CMRO_2$ values. Repeated-measures ANOVA followed by Bonferroni-corrected paired t-tests for absolute stimulus-induced $CMRO_2$ values. Bonferroni-corrected Wilcoxon signed-rank tests against baseline for normalized $CMRO_2$ values. n.s., not significant.

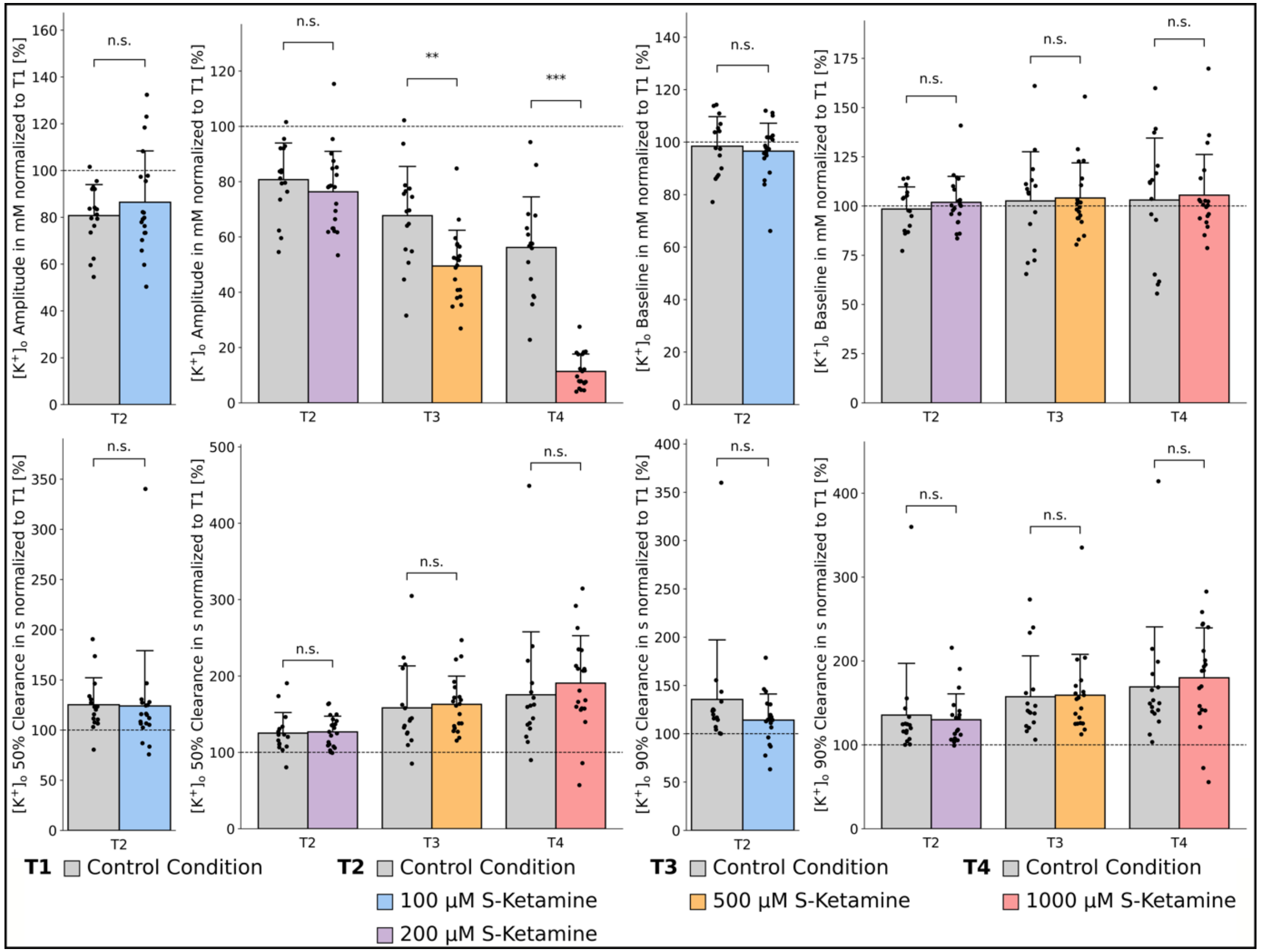

Supplementary Figure S3. Effects of 100–1000 µM S-ketamine on stimulus-induced potassium amplitude, clearance times, and baseline potassium levels. Concentrations are shown alongside the time-matched control condition without pharmacological intervention. In line with the observed changes in $CMRO_2$, both 500 and 1000 µM S-ketamine significantly reduced the potassium amplitude compared to the control condition (p = 0.003, Bonferroni-corrected Student's t-test, and $p < 0.001$, Bonferroni-corrected Mann–Whitney U test, respectively). No additional effects on potassium parameters were detected. Bar charts display mean + SD and individual data points. n = 15–20 slices per group from 3–7 animals. Statistical comparisons: Bonferroni-corrected Student's t-test, Welch's t-test, or Mann–Whitney U test, depending on data distribution and variance homogeneity. ** $p < 0.01$, *** $p < 0.001$; n.s., not significant.

2. Supplementary Results Tables

The following tables present sex-dependent data summaries and statistical analyses. All summary data are reported as median values with interquartile ranges (25th–75th percentiles, Weibull method). Rows corresponding to data from male mice are highlighted in blue, female mice in red, and combined data (both sexes) in green. *n* denotes the number of slices, and *N* denotes the number of animals.

Supplementary Table S1. Effects of 100 μM S-ketamine on basal and stimulus-induced $CMRO_2$ and ATP consumption rates, baseline potassium levels, stimulus-evoked potassium amplitudes, and potassium clearance times. Conditions: T1, control; T2, 100 μM S-ketamine. Statistical tests: 1, Wilcoxon signed-rank test; 2, paired t-test.

	T1	T2	*n*	*N*	p-Value	Test
Basal $CMRO_2$ (mmHg/s)	17.17 (14.48–20.35)	17.85 (15.24–22.02)	18	2	0.286	1
	24.07 (18.91–29.88)	23.94 (17.47–28.13)	4	1	0.429	2
	17.90 (14.80–21.97)	19.10 (15.83–24.39)	22	3	0.548	1
Basal Relative ATP consumption rate	0.93 (0.83–1.04)	0.95 (0.86–1.10)	18	2	0.286	1
	1.16 (0.99–1.33)	1.15 (0.94–1.28)	4	1	0.394	2
	0.96 (0.84–1.10)	1.00 (0.88–1.17)	22	3	0.548	1
Stimulus induced $CMRO_2$ (mmHg/s)	20.38 (16.66–22.85)	20.49 (16.08–25.55)	17	2	0.356	1
	30.30 (21.91–34.42)	27.08 (19.13–33.55)	4	1	0.053	2
	20.42 (17.53–25.79)	20.77 (16.95–27.79)	21	3	0.848	1
Stimulus induced relative ATP consumption rate	1.04 (0.91–1.12)	1.05 (0.89–1.21)	17	2	0.435	1
	1.34 (1.09–1.46)	1.25 (1.00–1.43)	4	1	0.053	2
	1.04 (0.94–1.22)	1.06 (0.92–1.27)	21	3	0.821	1
$[K+]_o$ Amplitude (mM)	1.17 (0.40–1.73)	1.23 (0.46–1.42)	15	2	0.016	2
	1.19 (0.82–2.98)	0.84 (0.52–1.94)	4	1	0.103	2
	1.17 (0.54–1.73)	0.93 (0.46–1.42)	19	3	0.003	2

$[K+]_o$ Baseline (mM)	2.99 (2.94–3.03)	2.86 (2.67–2.99)	15	2	0.050	2
	3.00 (2.96–3.01)	3.18 (2.98–3.30)	4	1	0.159	2
	2.99 (2.95–3.03)	2.89 (2.68–3.09)	19	3	0.126	1
$[K+]_o$ 50% Clearance (s)	2.64 (2.08–4.44)	2.81 (2.43–4.98)	15	2	0.200	2
	2.00 (1.70–2.59)	2.81 (1.80–5.91)	4	1	0.068	1
	2.54 (1.98–4.42)	2.81 (2.34–4.98)	19	3	0.040	1
$[K+]_o$ 90% Clearance (s)	9.29 (6.91–13.25)	9.07 (7.72–14.29)	14	2	0.300	1
	7.75 (7.19–8.44)	9.65 (7.31–14.13)	4	1	0.186	2
	8.58 (7.09–12.38)	9.08 (7.72–14.29)	18	3	0.078	1

Supplementary Table S2. Effects of 200–1000 µM S-ketamine on basal and stimulus-induced $CMRO_2$ and ATP consumption rates, baseline potassium levels, stimulus-evoked potassium amplitudes, and potassium clearance times. Conditions: T1, control; T2, 200 µM S-ketamine; T3, 500 µM S-ketamine; T4, 1000 µM S-ketamine. Statistical tests: 1, Wilcoxon signed-rank test (Bonferroni-corrected); 2, paired t-test (Bonferroni-corrected).

	**T1**	**T2**	**T3**	**T4**	**n**	**N**	**p-Values**			**Test**
							**T1 vs. T2**	**T1 vs. T3**	**T1 vs. T4**	
Basal $CMRO_2$ (mmHg/s)	37.94 (32.16–46.54)	40.21 (31.13–44.00)	41.53 (26.27–49.32)	35.85 (26.18–45.18)	8	3	1	1	0.484	1
	33.31 (30.23–38.47)	33.71 (29.79–40.61)	33.82 (26.12–36.68)	29.84 (23.66–33.59)	16	4	1	0.363	0.131	1
	35.24 (30.41–42.00)	35.19 (29.79–42.21)	35.35 (26.12–38.69)	32.40 (24.03–36.29)	24	7	1	0.276	0.042	1

Basal Relative ATP consumption rate	1.04 (0.93–1.19)	1.09 (0.91–1.15)	1.11 (0.81–1.24)	1.01 (0.81–1.17)	8	3	1	1	0.484	1
	0.96 (0.90–1.05)	0.97 (0.89–1.09)	0.97 (0.81–1.02)	0.89 (0.76–0.96)	16	4	1	0.294	0.09	1
	1.00 (0.90–1.12)	0.99 (0.89–1.12)	1.00 (0.81–1.06)	0.94 (0.76–1.01)	24	7	1	0.229	0.028	1
Stimulus induced $CMRO_2$ (mmHg/s)	41.46 (36.18–59.82)	45.45 (33.54–52.63)	45.40 (27.27–54.79)	36.11 (26.56–45.74)	8	3	1	0.648	0.032	2
	40.55 (32.84–49.31)	40.93 (33.37–51.22)	37.01 (28.99–43.21)	31.02 (23.93–35.65)	16	4	0.536	0.029	0.005	1
	41.27 (33.30–52.08)	43.16 (33.37–51.32)	39.13 (28.99–45.68)	32.87 (24.45–40.71)	24	7	0.390	0.013	< 0.001	1
Stimulus induced relative ATP consumption rate	1.11 (1.01–1.40)	1.18 (0.96–1.29)	1.17 (0.83–1.32)	1.01 (0.82–1.18)	8	3	1	0.56	0.022	2
	1.09 (0.95–1.24)	1.10 (0.96–1.27)	1.03 (0.87–1.14)	0.91 (0.76–1.00)	16	4	0.536	0.029	0.003	1
	1.10 (0.96–1.28)	1.14 (0.96–1.27)	1.07 (0.87–1.18)	0.95 (0.77–1.09)	24	7	0.369	0.013	< 0.001	1
$[K+]_o$ Amplitude (mM)	2.21 (0.70–3.04)	1.73 (0.59–2.38)	1.16 (0.36–1.52)	0.16 (0.05–0.26)	7	3	0.083	0.021	0.013	2
	3.31 (1.64–4.37)	2.38 (1.08–3.91)	1.52 (0.65–3.00)	0.40 (0.14–0.82)	13	4	0.009	< 0.001	< 0.001	2
	2.53 (1.53–3.96)	1.92 (1.05–3.62)	1.33 (0.65–2.18)	0.22 (0.13–0.45)	20	7	0.001	< 0.001	< 0.001	1

$[K+]_o$ Baseline (mM)	3.00 (2.99–3.01)	2.98 (2.94–3.46)	3.09 (2.95–3.68)	3.01 (3.00–3.08)	7	3	1	0.931	0.931	1
	3.01 (2.94–3.02)	2.98 (2.61–3.28)	2.92 (2.58–3.37)	3.02 (2.66–3.49)	13	4	1	1	1	1
	3.00 (2.98–3.02)	2.98 (2.80–3.28)	3.01 (2.80–3.38)	3.01 (2.80–3.36)	20	7	1	1	1	1
$[K+]_o$ 50% Clearance (s)	2.16 (1.50–4.01)	2.55 (1.78–5.01)	3.22 (2.33–6.02)	3.68 (3.15–4.93)	7	3	0.128	0.054	0.273	1
	1.98 (1.65–2.83)	2.64 (1.91–3.73)	3.16 (2.24–4.82)	3.82 (2.75–5.59)	13	4	0.001	< 0.001	< 0.001	2
	2.03 (1.63–2.95)	2.60 (1.88–3.88)	3.19 (2.27–4.92)	3.75 (2.87–5.13)	20	7	< 0.001	< 0.001	0.002	1
$[K+]_o$ 90% Clearance (s)	7.27 (4.87–11.60)	7.83 (6.19–12.33)	9.97 (7.24–16.28)	11.84 (9.49–17.47)	7	3	0.054	0.054	0.385	1
	7.70 (6.59–8.77)	8.92 (7.53–12.50)	11.72 (8.38–16.06)	12.88 (9.33–21.66)	13	4	0.006	0.004	0.006	1
	7.70 (6.34–9.53)	8.54 (7.39–12.33)	11.17 (8.32–16.09)	12.36 (9.52–19.13)	20	7	< 0.001	< 0.001	0.002	1

Supplementary Table S3. Effects of 100 µM S-ketamine on basal $CMRO_2$ and ATP consumption rates, gamma frequency, and gamma power. Conditions: T1, control; T2, 150 nM kainate (gamma oscillations); T3, 150 nM kainate (gamma oscillations) + 100 µM S-ketamine. Statistical tests: 1, Wilcoxon signed-rank test (Bonferroni-corrected); 2, paired t-test (Bonferroni-corrected).

	T1	T2	T3	n	N	p-Values			Test
						T1 vs. T2	T1 vs. T3	T2 vs. T3	
Basal $CMRO_2$ (mmHg/s)	27.59 (21.61–32.45)	41.24 (29.56–44.80)	38.62 (29.04–43.54)	12	2	0.007	0.007	0.084	1
	25.05 (16.18–30.17)	35.52 (28.13–37.54)	29.95 (22.74–34.11)	11	3	0.010	0.038	0.010	1
	26.84 (20.44–30.17)	35.99 (29.08–44.14)	32.61 (27.55–42.30)	23	5	< 0.001	< 0.001	0.001	1
Basal Relative ATP consumption rate	1.06 (0.90–1.18)	1.38 (1.11–1.45)	1.32 (1.10–1.42)	12	2	0.007	0.007	0.084	1
	1.00 (0.74–1.13)	1.25 (1.08–1.30)	1.12 (0.93–1.22)	11	3	0.010	0.049	0.010	1
	1.04 (0.87–1.13)	1.26 (1.10–1.43)	1.18 (1.06–1.40)	23	5	< 0.001	< 0.001	0.001	1
Gamma Frequency (Hz)		36.01 (31.74–39.98)	26.86 (24.41–28.99)	12	2			<0.001	2
		34.18 (31.74–35.40)	24.41 (23.19–26.86)	11	3			<0.001	2
		34.18 (31.74–39.06)	25.63 (24.41–28.08)	23	5			<0.001	2
Gamma Power ($*10^{-3} mV^2$)		0.11 (0.02–0.39)	0.14 (0.02–0.42)	12	2			0.209	1
		0.16 (0.01–1.21)	0.25 (0.01–0.80)	11	3			0.790	1
		0.12 (0.02–0.54)	0.19 (0.01–0.48)	23	5			0.616	1

Supplementary Table S4. Effects of 100 µM S-ketamine on basal $CMRO_2$ and ATP consumption rates, seizure-like event frequency, and interictal-like event frequency. Conditions: T1, control; T2, 0 mM Mg^{2+}; T3, 0 mM Mg^{2+} + 100 µM S-ketamine. Statistical tests: 1, Wilcoxon signed-rank test (Bonferroni-corrected); 2, paired t-test (Bonferroni-corrected).

	T1	T2	T3	n	N	p-Values			Test
						T1 vs. T2	T1 vs. T3	T2 vs. T3	
Basal $CMRO_2$ (mmHg/s)	22.60 (17.98–28.01)	25.44 (20.50–37.00)	21.24 (17.62–28.97)	17	7	< 0.001	1	< 0.001	1
	21.49 (20.00–26.81)	26.38 (23.72–32.00)	23.98 (20.31–25.06)	5	4	0.003	1	0.121	2
	22.05 (18.58–27.91)	25.91 (21.48–35.90)	22.61 (18.07–26.91)	22	11	< 0.001	1	< 0.001	1
Basal Relative ATP consumption rate	0.98 (0.84–1.13)	1.06 (0.92–1.35)	0.94 (0.83–1.16)	17	7	< 0.001	1	< 0.001	1
	0.95 (0.91–1.10)	1.09 (1.02–1.24)	1.02 (0.91–1.05)	5	4	0.003	1	0.101	2
	0.97 (0.86–1.13)	1.08 (0.95–1.33)	0.98 (0.84–1.10)	22	11	< 0.001	1	< 0.001	1
Seizure Like Events (count / 10min)		4.00 (2.00–5.00)	0.00 (0.00–0.00)	11	4			<0.001	2
		2.00 (1.00–4.50)	0.00 (0.00–0.00)	4	3			0.08	2
		3.00 (1.00–5.00)	0.00 (0.00–0.00)	15	7			<0.001	1
Interictal Like Events (count / 10min)		21.50 (10.75–68.25)	0.00 (0.00–5.25)	14	7			<0.001	1
		48.00 (24.75–82.50)	1.00 (0.00–10.25)	4	3			0.035	2
		28.00 (15.25–68.25)	0.00 (0.00–4.75)	18	10			<0.001	1

Supplementary Table S5. Effects of 100 and 1000 µM S-ketamine on the oxidative peak, reductive undershoot, and FAD decay. Groups: G1, control; G2, 100 µM S-ketamine; G3, 1000 µM S-ketamine. Statistical tests: 1, Tukey's HSD; 2, Games–Howell; 3, Dunn's test (Bonferroni-corrected).

	**G1**	**n**	**N**	**G2**	**n**	**N**	**G3**	**n**	**N**	**p-Values**			**Test**
										**G1 vs. G2**	**G1 vs. G3**	**G2 vs. G3**	
Oxidative Peak ($\Delta F/F_0$)	3.48 (2.74–3.77)	10	3	2.63 (1.38–3.55)	7	1	1.16 (0.80–1.37)	7	1	0.237	< 0.001	0.022	1
	2.32 (1.94–3.03)	12	5	2.65 (2.33–3.58)	15	5	0.91 (0.31–1.13)	15	4	1	0.018	< 0.001	3
	2.80 (2.26–3.51)	22	8	2.64 (2.09–3.56)	22	6	0.97 (0.32–1.35)	22	5	1	< 0.001	< 0.001	3
Reductive Undershoot ($\Delta F/F_0$)	-4.74 (-5.32–-3.86)	10	3	-3.37 (-4.15–-3.13)	7	1	-1.54 (-2.10–-1.22)	7	1	0.015	< 0.001	< 0.001	1
	-3.67 (-4.02–-3.01)	12	5	-3.51 (-4.08–-2.60)	15	5	-0.98 (-2.15–-0.47)	15	4	1	< 0.001	0.001	3
	-3.98 (-5.00–-3.45)	22	8	-3.39 (-4.10–-2.70)	22	6	-1.19 (-2.11–-0.53)	22	5	0.459	< 0.001	< 0.001	3
Decay (% / min)	-1.73 (-2.26–-1.31)	10	3	-1.25 (-1.45–-0.98)	7	1	-2.97 (-3.09–-2.22)	7	1	0.063	< 0.001	< 0.001	1
	-1.43 (-2.07–-1.24)	12	5	-1.55 (-1.75–-1.44)	15	5	-2.76 (-3.06–-1.52)	15	4	0.903	0.008	0.002	2
	-1.52 (-2.21–-1.29)	22	8	-1.46 (-1.65–-1.14)	22	6	-2.77 (-3.07–-1.81)	22	5	0.165	< 0.001	< 0.001	2

Supplementary Table S6. Comparison of basal and stimulus-induced $CMRO_2$ and ATP consumption rates, baseline potassium levels, stimulus-evoked potassium amplitudes, and potassium clearance times in slices without pharmacological intervention across time points T1–T4. Conditions: T1, control; T2, control; T3, control; T4, control. Statistical tests: 1, Wilcoxon signed-rank test (Bonferroni-corrected); 2, paired t-test (Bonferroni-corrected).

	T1	T2	T3	T4	n	N	p-Values			Test
							T1 vs. T2	T1 vs. T3	T1 vs. T4	
Basal $CMRO_2$ (mmHg/s)	21.98 (18.72–30.33)	21.06 (20.05–35.54)	21.09 (17.44–38.67)	24.45 (21.43–36.44)	8	2	1	1	0.623	1
	33.49 (27.00–49.84)	33.78 (27.51–50.96)	36.32 (29.32–48.72)	37.12 (28.70–47.86)	10	4	1	0.486	1	2
	29.85 (20.31–40.93)	30.69 (20.79–43.23)	33.65 (18.79–43.88)	31.73 (24.07–41.09)	18	6	1	1	0.693	1
Stimulus induced $CMRO_2$ (mmHg/s)	27.79 (24.60–34.37)	27.72 (24.60–41.23)	24.11 (20.71–42.99)	27.90 (25.03–38.11)	8	2	1	0.788	1	1
	46.22 (29.29–66.12)	46.29 (30.12–67.45)	47.07 (32.21–65.03)	47.94 (30.87–64.31)	10	4	1	1	1	2
	33.31 (24.95–59.55)	37.03 (24.68–62.05)	38.76 (22.80–60.84)	37.56 (26.44–57.38)	18	6	1	1	1	2
$[K+]_o$ Amplitude (mM)	3.11 (2.34–4.41)	2.49 (1.31–3.77)	1.94 (0.89–3.10)	1.74 (0.68–2.75)	8	2	0.005	0.002	0.001	2
	4.76 (1.06–6.65)	3.89 (0.78–5.49)	3.27 (0.56–4.71)	2.28 (0.45–3.76)	8	4	0.072	0.046	0.034	2
	3.30 (1.67–5.20)	2.92 (1.00–4.02)	2.43 (0.78–3.63)	1.88 (0.63–3.12)	16	6	< 0.001	< 0.001	< 0.001	2

$[K^+]_o$ Baseline (mM)	3.02 (2.96–3.03)	3.13 (2.97–3.41)	3.25 (2.99–3.53)	3.34 (2.92–3.58)	8	2	0.553	0.978	1	2
	3.00 (2.95–3.02)	2.73 (2.58–2.86)	2.74 (2.17–3.31)	2.81 (1.85–4.18)	7	4	0.122	1	1	2
	3.01 (2.95–3.02)	2.95 (2.73–3.15)	3.19 (2.35–3.38)	3.28 (1.98–3.63)	15	6	1	1	1	2
$[K^+]_o$ 50% Clearance (s)	3.65 (2.11–4.74)	4.15 (2.45–6.03)	4.86 (2.86–7.71)	5.28 (3.03–7.93)	8	2	0.035	0.035	0.035	1
	1.47 (1.34–2.56)	1.85 (1.45–3.03)	2.14 (1.71–4.48)	2.39 (1.81–4.20)	8	4	0.206	0.107	0.075	1
	2.40 (1.45–3.70)	2.88 (1.84–5.23)	3.64 (2.06–6.40)	3.80 (2.38–6.79)	16	6	0.006	0.002	0.002	1
$[K^+]_o$ 90% Clearance (s)	12.15 (8.46–15.05)	14.88 (9.76–19.07)	19.02 (11.33–22.79)	20.03 (12.62–23.54)	8	2	0.035	0.035	0.035	1
	6.26 (4.51–8.56)	6.98 (4.92–13.69)	9.87 (5.64–16.78)	8.74 (6.02–15.06)	8	4	0.035	0.035	0.035	1
	8.24 (6.04–12.30)	11.69 (6.50–17.90)	14.99 (7.35–21.31)	14.37 (8.32–22.94)	16	6	0.001	0.001	0.001	1
